# '*We pledge to improve the health of our entire community*': Improving health worker motivation and performance in Bihar, India through teamwork, recognition, and non-financial incentives

**DOI:** 10.1371/journal.pone.0203265

**Published:** 2018-08-30

**Authors:** Carolyn Grant, Dipty Nawal, Sai Mala Guntur, Manish Kumar, Indrajit Chaudhuri, Christine Galavotti, Tanmay Mahapatra, Kunal Ranjan, Gangesh Kumar, Sunil Mohanty, Mohammed Aftab Alam, Aritra Das, Safia Jiwani

**Affiliations:** 1 Sexual and Reproductive Health and Rights, CARE USA, Atlanta, Georgia, United States of America; 2 CARE India Solutions for Sustainable Development, Patna, Bihar, India; 3 Jeevika Technical Support Program, Project Concern International, Patna, Bihar, India; 4 Department of International Health, Johns Hopkins Bloomberg School of Public Health, Baltimore, Maryland, United States of America; ESIC Medical College & PGIMSR, INDIA

## Abstract

**Background:**

Motivation is critical to health worker performance and work quality. In Bihar, India, frontline health workers provide essential health services for the state’s poorest citizens. Yet, there is a shortfall of motivated and skilled providers and a lack of coordination between two cadres of frontline health workers and their supervisors. CARE India developed an approach aimed at improving health workers’ performance by shifting work culture and strengthening teamwork and motivation. The intervention—“Team-Based Goals and Incentives”—supported health workers to work as teams towards collective goals and rewarded success with public recognition and non-financial incentives.

**Methods:**

Thirty months after initiating the intervention, 885 health workers and 98 supervisors completed an interviewer-administered questionnaire in 38 intervention and 38 control health sub-centers in one district. The questionnaire included measures of social cohesion, teamwork attitudes, self-efficacy, job satisfaction, teamwork behaviors, equitable service delivery, taking initiative, and supervisory support. We conducted bivariate analyses to examine the impact of the intervention on these psychosocial and behavioral outcomes.

**Results:**

Results show statistically significant differences across several measures between intervention and control frontline health workers, including improved teamwork (mean = 8.8 vs. 7.3), empowerment (8.5 vs. 7.4), job satisfaction (7.1 vs. 5.99) and equitable service delivery (6.7 vs. 4.99). While fewer significant differences were found for supervisors, they reported improved teamwork (8.4 vs. 5.3), and frontline health workers reported improved fulfillment of supervisory duties by their supervisors (8.9 vs. 7.6). Both frontline health workers and supervisors found public recognition and enhanced teamwork more motivating than the non-financial incentives.

**Conclusions:**

The Team-Based Goals and Incentives model reinforces intrinsic motivation and supports improvements in the teamwork, motivation, and performance of health workers. It offers an approach to practitioners and governments for improving the work environment in a resource-constrained setting and where there are multiple cadres of health workers.

## Introduction

Motivation is considered critical to health worker retention and performance, as well as for other key outcomes important to work quality [1–4]. Improving health worker motivation has also been recognized as essential to achieving national and global health goals in low income countries and universal health coverage [1, 3, 4]. In the work context, motivation is defined as: an individual's degree of willingness to exert and maintain an effort towards attaining organizational goals [5]. Researchers describe worker motivation as a complex set of psychological processes that involve both intrinsic and extrinsic factors [5, 6] and suggest that it is a result of interactions between individual, organizational, and cultural determinants [2, 7–11]. Evidence also tells us that interventions to support motivation are associated with three major outcomes: cognitive aspects (job attachment), affection (job satisfaction), and behavioral outcomes (job performance)—in other words, motivation affects what workers think, feel, and do [2, 5, 9, 11]. While several studies have reported on the determinants of health worker motivation, there are fewer studies that have explored the conceptualization or evaluated the impacts of interventions aimed at improving health worker motivation in low-income countries [1, 2, 7, 11–13].

In India, frontline health workers (FLHWs) are the cornerstones of the Government of India’s Reproductive, Maternal, Newborn, Child, and Adolescent Health (RMNCH+A) strategy for delivering services to the country’s population, especially in rural areas [14]. India’s FLHWs serve as vital links between the community and the formal health system and consist of the following three cadres, all female: Anganwadi Workers (AWW), Accredited Social Health Activists (ASHA), and Auxiliary Nurse Midwives (ANM) [10, 13, 15]. The delivery of RMNCH+A services through FLHWs are critical in states like Bihar, one of India’s largest states, where 88.71 percent of the population lives in rural areas [16]. While Bihar has seen significant improvements in the health and well‐being of its population in recent years, it still has some of the country’s highest rates of infant and child mortality and lowest maternal health indicators, as well as a high prevalence of malnutrition, anemia, stunted growth, and high fertility rates [17]. Bihar has also struggled with a very resource-constrained health system faced with inadequate public health infrastructure, supplies, and equipment and an acute shortage of skilled providers, including FLHWs [18].

India’s FLHWs face numerous challenges, including: poor treatment by the officials and the communities they serve; lack of trust, teamwork, and coordination between the three cadres of FLHWs, even though they are supposed to be working together; unreliable and inadequate payment; weak training; and poor job planning, supervision, monitoring, and accountability [10, 18–20]. The ASHAs, who are volunteers paid on an incentive-based scheme, face the additional challenge of expectations to provide services and perform work duties for which they are not directly compensated or recognized. These additional duties include facilitating village health meetings, counseling on non-incentivized practices, and acting as health activists in the community [10, 19, 20].

To address the work environment challenges faced by Bihar’s FLHWs, CARE India designed and tested an intervention focused on increasing the motivation and teamwork of the FLHWs. The goal of the intervention, called ‘Team-Based Goals and Incentives’ (TBGI), was to improve the performance of FLHWs and the coverage, quality, and equity of RMNCH+A services they deliver. We hypothesized that FLHWs (ASHAs, AWWs, and ANMs), who worked in teams to achieve collective goals and received rewards and recognition for their success, would be more motivated and would demonstrate improved performance outcomes compared to FLHWs who worked without these incentives and shared targets. This paper describes the conceptualization and design of CARE’s TBGI intervention and assesses its effect on the teamwork, motivation, and performance of Bihar’s FLHWs.

## Background

### Intervention design and conceptual model

The conceptualization and design of the TBGI intervention drew on *Employee motivational theory* [6] and health worker motivation literature [2, 5, 8, 9, 21], as well as on existing research on the work environment challenges and motivational determinants for FLHWs in India, and in Bihar specifically, where available [7, 18, 19]. Resulting from this research, we considered the following determinants of motivation in the design of the intervention: self-efficacy to do one’s job; personal goals and values; recognition and appreciation; opportunities for training, advancement, and growth; supportive supervision and leadership; relationships at work and team climate; and availability of appropriate resources and inputs to do one’s job. While both financial and non-financial factors have been found to influence health worker motivation, CARE decided to test the power of non-financial incentives on motivation and performance. Other studies in Andhra Pradesh and Uttar Pradesh, India, which found that ‘good income’ was not rated in the top ten most important characteristics of motivation, but was, in fact, the third least important factor [7], supported this emphasis on non-financial incentives.

**Fig 1:**
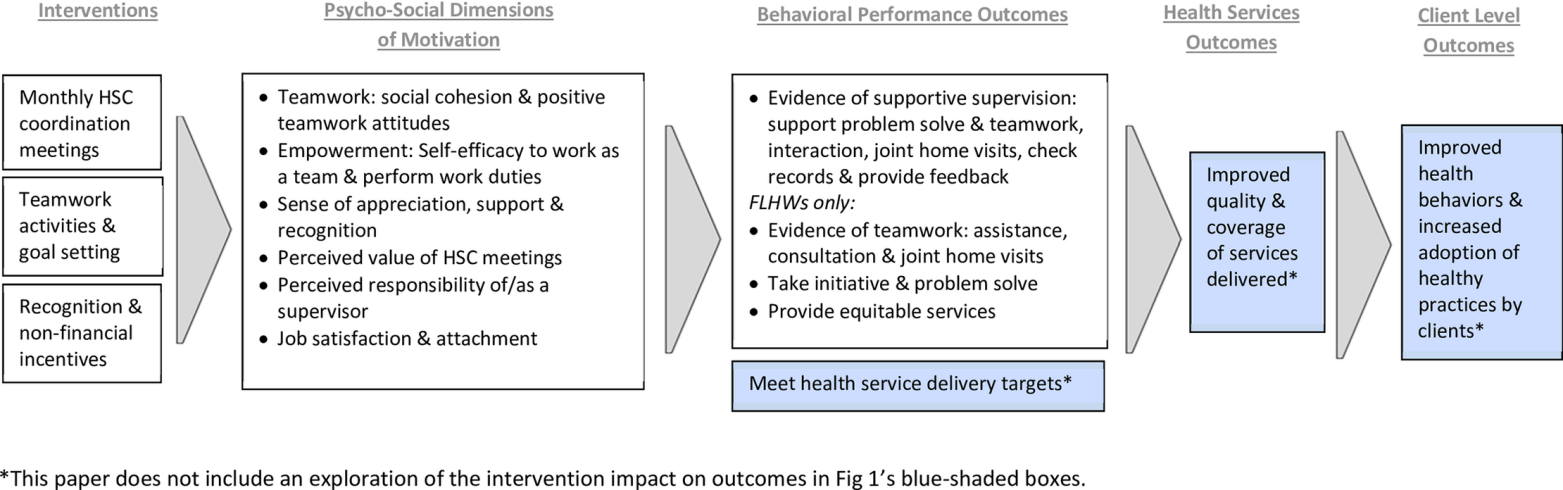
Team-Based Goals and Incentives conceptual model.

The TBGI conceptual model (Fig 1) describes how we expected the TBGI intervention components to improve FLHW teamwork, motivation, and performance and, in turn, lead to higher quality and increased coverage of RMNCH+A services delivered, thus, eventually, improving the health behaviors and practices among clients.

The conceptual model details the psychosocial dimensions of motivation we expected the TBGI intervention to impact positively, including social cohesion and positive attitudes towards teamwork, self-efficacy to work as a team and perform work duties, sense of appreciation and recognition, and job satisfaction and attachment. We also expected to see improvements in FLHWs’ perceived value of the Health Sub-Center meeting (an organizational input to support FLHWs in doing their jobs) and their perceived responsibility of supervisor (as a measure for supervisory support). Expected behavioral performance outcomes included: enhanced teamwork, as evidenced by increased interaction, consultation, and joint-home visits; improvements in taking initiative and problem solving when confronted with obstacles; and the consistent meeting of health service delivery targets. We also expected to see FLHWs visiting more marginalized communities, indicated in the conceptual model as ‘provides more equitable services’. Finally, the intervention expected to observe improvements in the supervisory support provided by the ANMs to the ASHAs and AWWs.

The resulting intervention design consisted of three main components: 1) monthly coordination meetings among all the FLHWs, including the ANM who plays a supervisory role; 2) teamwork activities and goal setting; and 3) non-financial incentives and recognition.

#### Monthly coordination meetings

CARE India initiated a monthly Health Sub-Center (HSC) meeting that brought together the FLHWs working in the same catchment area for joint review, planning, and coordination of service delivery, as well as for teamwork activities. All the ASHAs and AWWs working in a single HSC catchment area and their overseeing ANM constituted a single TBGI team.

The HSC meetings were also used as a platform for conducting capacity training, including counseling and leadership skills, as well as training for the ANMs to improve their supervisory support and capacity to run the monthly meetings. For the purposes of this study, from here forward, the classification of FLHWs refers specifically to the ASHAs and AWWs and the classification of supervisors refers to the ANMs.

#### Teamwork activities and goal setting

The teamwork activities consisted of a mix of team building and motivational activities aimed at shifting the work culture and fostering solidarity and collaboration within each team. These included: 1) joint setting of and monitoring and reporting on service delivery goals; 2) joint development of team norms; 3) recitation of a motivational team pledge at the monthly meetings; and 4) joint home visits.

CARE set the service delivery goals–percent coverage–for a range of RMNCH+A services provided at the HSC- and household-levels in consultation with FLHWs and supervisors (Table 1). We trained the FLHWs how to convert the coverage goals, using simple calculations, into a target number of households, women, or children they had to reach in each village per month. Each team had autonomy to determine how they would achieve the targets. We provided the FLHWs with diaries to track their progress. The diaries also included a team picture, basic guidelines on the goals, the team norms which they set in consultation with each other, and the motivational pledge. At the monthly meetings, the FLHWs reported on their progress in achieving the goals to their supervisor and jointly discussed any issues. We also encouraged the FLHWs to conduct joint home visits and to consult each other and their supervisors proactively when they encountered problems.

**Table 1:** TBGI service delivery targets, the ‘Goals’.

Service Delivery Target	% coverage of total relevant population served
MATERNAL HEALTH	
Pregnant women registered for antenatal care (ANC) during 1st trimester	70%
Pregnant women receiving at least 3 ANC check-ups	80%
Pregnant women receiving tetanus toxoid-2 or booster	90%
Pregnant women have a transportation plan for normal & emergency delivery	90%
Mothers received at least 90 Iron/Folic Acid tablets during their last pregnancy	80%
Institutional deliveries	80%
NEW BORN HEALTH	
Home-delivered newborns visited within 24hrs of delivery	80%
Newborns breastfed within 1 hour of delivery	90%
Deliveries following clean cord practice	90%
NUTRITION	
Children receiving age appropriate frequency of complementary feeding between 6–11 months of age	70%
Children receiving age-appropriate quantity of complementary feeding between 6–11 months of age	70%
FAMILY PLANNING	
Mothers adopting a modern method of family planning within 6 months of delivery	40%
IMMUNIZATION	
Children receiving DPT3 by 6 months	90%
Infants 0 to 11 months old receiving measles vaccine	90%
REFERRAL	
Complications during pregnancy/postnatal identified and referred	N/A
High-risk neonates identified, cared for, and referred	N/A
Sepsis cases identified, cared for, and referred	N/A

#### Non-financial incentives and recognition

On a quarterly basis, each team submitted a report to the Block Health Manager and medical officers on their progress toward achieving the service delivery targets. These officials, who were stakeholders of the health programs that employed the FLHWs, verified five percent of the reports quarterly by visiting a selection of the client households. Successful teams, those which achieved of 12 out of 17 targets, received a non-financial reward. The non-financial rewards consisted of household items that the teams identified as valuable. Those teams that on average achieved 12 of 17 targets across all four quarters received a certificate of recognition, signed and awarded by the Block Health Managers during a public ceremony in the community.

## Methods

### Study design and sampling

We conducted this study as a follow-on to a cluster randomized control trial (RCT) that was focused on the TBGI intervention’s impact on the quality of the FLHWs’ home visits and on beneficiary-level RMNCH+A knowledge and health outcomes [22]. The objective of our study was to evaluate the impact of the TBGI intervention on teamwork as well as on the motivation and performance of both the FLHWs and supervisors.

In designing our follow-on study, we followed the RCT randomization procedure and cluster selection. The intervention and original RCT were focused in five blocks (of 18 total blocks) in Begusarai district of Bihar, which were selected to represent a range of sizes and geographies. Blocks that CARE’s team had qualitatively determined to be atypical, such as those in which government health officials were not in place, were excluded. All the health subcenters (76 in total) in the selected blocks were randomly assigned into equal-sized treatment and control groups (38 subcenters in each group) using a stratified random assignment procedure based on the number of Anganwadi Centers (AWCs) served by the subcenter (a proxy for the size of the population served). The stratification helped to ensure that the treatment and control groups were balanced by the size of the population served. We then conducted a census, interviewing all the FLHWs (ASHAs and AWWs) and supervisors (ANMs) working in the 76 selected HSCs at the time of data collection.

The ‘Institutional Committee for Ethics and Review of Research’ of Indian Institute of Health Management Research (www.iihmr.org), Jaipur, India reviewed and approved the study protocol and procedures. Consent from study participants was obtained verbally as per the approved protocol. The original trial was registered at clinicaltrials.gov (NCT03561012).

### Data collection

We developed different multi-item questionnaires for the FLHWs and the supervisors to assess the influence of TBGI on the variables of the conceptual model. We first pilot tested the questionnaires with a small cohort of FLHWs and supervisors and then used the results to refine the survey tools further. Trained CARE evaluation staff administered the finalized questionnaires in face to face interviews with all of the ASHAs and AWWs (n = 885) and ANMs (n = 98) across all 76 HSCs. The data collection took place 30 months after initiation of the intervention.

### Measure development

We developed and adapted the questionnaires from validated tools available in the literature on measuring health worker motivation [2, 7, 12, 23] and drew on relevant CARE measurement tools [24, 25]. As none of the existing validated tools in their pure form seamlessly aligned with the outcomes we sought to measure per our theory of change, we compiled and adapted from these multiple sources. The measures we arrived at and the steps taken to validate are detailed in the next sections.

#### Psychosocial dimensions of motivation

We developed six measures, each consisting of several items, to assess the psychosocial dimensions of motivation outlined in our conceptual model. The teamwork measure explored the constructs of social cohesion, such as trust, respect, and rapport among team members, as well as teamwork attitudes, such as belief that working as a team makes one’s job easier. We assessed empowerment in the workplace with questions on self-efficacy to work as a team, express one’s opinion, and perform work duties. To measure sense of appreciation, support, and recognition, we asked about respect and support from family, community, and colleagues. We also posed a single question around reliable payment. We incorporated several questions to measure the perceived value of the monthly coordination meetings. To evaluate supervisory support, we included questions on perceived responsibility of and as a supervisor, respectively, such as ‘it is your/your ANM’s responsibility to resolve conflicts, communicate a clear vision, or foster inclusiveness’. Finally, job satisfaction and attachment included questions for satisfaction with work, opportunity to use abilities and learn new things, as well as questions on ‘belief in mission’, ‘only doing the job for money’ and ‘would leave if found another job’.

#### Behavioral performance outcomes

We developed measures for all four behavioral outcomes outlined in our conceptual model for the FLHWs. We assessed supervisors on supportive supervision only. To evaluate evidence of teamwork, we included questions on ‘helping each other’ and sharing information, as well as on frequency of interaction, joint home visits, and HSC meeting attendance. We assessed equitable service delivery by asking FLHWs how often they visited the homes of Dalit women, a marginalized demographic in rural Bihar. To determine if FLHWs were more proactively taking initiative and problem solving, we included questions like, ‘You take initiative immediately, even when others do not’ and ‘You do things that need your attention without being asked or told to do so’. To better understand the initiative and teamwork constructs, we developed six scenario questions that asked the FLHWs what they would do in various situations, such as: ‘You visit a mother to counsel on complementary feeding, but her mother-in-law disagrees with you’. Response options included: doing nothing (scored as 0); a few different problem-solving actions they could take such as returning again later or talking to the husband (scored as 1); and an option that involved engaging another FLHW to help them with the situation, indicating teamwork (scored as 2).

To determine evidence of supportive supervision, we asked the ANMs about the support they provide to FLHWs and frequency of interaction. We included three scenario questions, such as, ‘An ASHA/AWW in your sub-center is not doing her work properly’. Response options for these scenarios included: doing nothing or scolding (scored as 0); indirect support or passive advising, like telling the FLHW to try again (scored as 1); and direct support or encouraging teamwork, such as accompanying the FLHW to the next home visit (scored as 2). We also asked FLHWs about fulfillment of supervisory duties to assess supervisory performance further, including the checking of records, observing home visits, and providing feedback. In the intervention HSC sites only, we asked all respondents about the value they placed on the different components of the intervention, including the non-financial incentives.

#### Measure validation and scoring

Following data collection, we reviewed distribution of responses on each item, eliminating those items with no variability. Most of the responses for our measures were captured using Likert-type scales, thus we calculated Cronbach’s alpha to assess domain-specific internal consistency and reliability. We considered Cronbach’s alpha of .60 to be an indication of acceptable reliability; and we considered .70 or higher to show high reliability. When measures did not perform well as scales, we kept the questions as single items in the analysis. We created indexes for all frequency items. We then constructed item, scale, and index scores, with high scores indicating positive responses. Finally, for ease of interpretation and comparison, we rescaled several measures on a scale of 10 as follows:

Construct=(TotalScoreReceived÷MaximumTotalScore)×10

Where,

$$\begin{array}{l} Total\;Score\;Received\\ \;=\left(Total\;number\;of\;responses\;of\;strongly\;agree\times 2\right)\\ \;+\left(Total\;number\;of\;responses\;of\;agree\times 1\right)\\ \;+\left(Total\;number\;of\;responses\;of\;disagree\times \left(-1\right)\right)\\ \;+\left(Total\;number\;of\;responses\;strongly\;disagree\times \left(-2\right)\right) \end{array}$$

**Fig 2:**
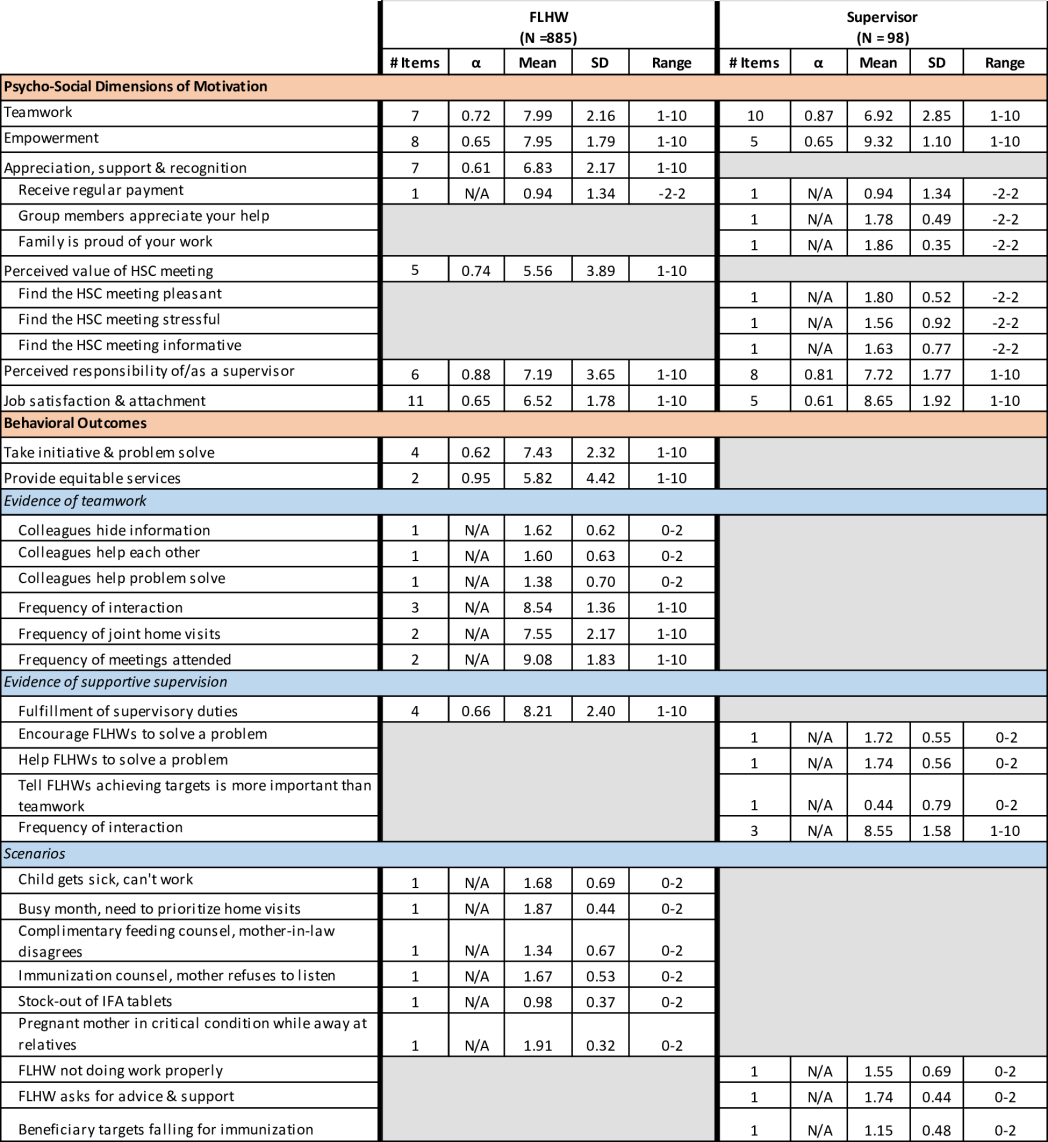
Psychometric properties of measures.

The description and psychometric properties of the measures are presented in Fig 2.

### Statistical analysis

We used chi-square and Fisher’s exact test to assess differences in socio-demographic characteristics between treatment and control groups, and we conducted separate bivariate analyses for both the FLHW and supervisor groups to estimate the impact of the intervention on psychosocial and behavioral performance outcomes. We evaluated differences in domain and item-wise mean scores between intervention and control groups using the Student’s t-test.

## Results

### FLHW and supervisor characteristics

Table 2 depicts the characteristics of the FLHW and supervisor sample. Overall, the supervisors tended to be older, from higher castes, and had more years of service than the FLHWs. There were no significant differences in characteristics between the intervention and control supervisors. Among the FLHWs, the intervention group had more FLHWs than the control group from either the general caste or scheduled caste/tribe (rather than from the backward caste) and who had been in their job for 5–10 years, but the differences were relatively small.

**Table 2:** Frontline health worker and supervisor characteristics.

	FLHW					Supervisor				
* *	Intervention (N = 428)		Control (N = 457)		Chi 2 P Value	Intervention (N = 52)		Control (N = 46)		Fisher exact P Value
	N	%	N	%		N	%	N	%	
**Health worker cadre**										
AWW	194	45.3	214	46.8	0.655					
ASHA	234	54.7	243	53.2						
ANM						52	100.0	46	100.0	N/A
**Age**										
≤ 30yrs	69	16.1	95	20.8	0.061	8	15.4	6	13.0	0.951
≥ 31 yrs and ≤ 40 yrs	260	60.8	243	53.2		8	15.4	7	15.2	
> 40 yrs	99	23.1	119	26.0		36	69.2	33	71.7	
**Education**										
Secondary Education (< = 10y)	184	43.0	211	46.2	0.412	20	38.5	14	30.4	0.318
Higher Secondary Education (11-12y)	160	37.4	171	37.4		24	46.2	28	60.9	
Graduation and above (>12y)	84	19.6	75	16.4		8	15.4	4	8.7	
**Caste**										
General Caste including others	156	36.5	150	32.8	0.004	33	63.5	28	60.9	0.837
Backward Caste	203	47.4	244	53.4		19	36.5	18	39.1	
Scheduled Caste and Scheduled Tribe	69	16.1	63	13.8		-	-	-	-	
**Marital status**										
Married	399	93.2	423	92.6	0.585	50	96.2	41	89.1	0.248
Never married/ widowed/divorced	29	6.8	34	7.4		2	3.9	5	10.9	
**Length of service**										
< = 5 yrs	68	15.9	99	21.7	0.012	3	5.8	4	8.7	0.902
>5 < = 10 yrs	276	64.5	251	54.9		20	38.5	17	37.0	
> 10 yrs	84	19.6	107	23.4		29	55.8	25	54.4	

### Intervention effect

Results for FLHWs showed a significant intervention impact on all psychosocial and behavioral measures, except for the ‘Take initiative & problem solve’ scale and two scenario items (Table 3).

**Table 3:** Intervention effect on FLHWs.

	Intervention (N = 428)		Control (N = 475)		P value
	Mean	CI	Mean	CI	
**Psychosocial Dimensions of Motivation**					
Teamwork	8.79	8.63–8.94	7.25	7.04–7.47	<0.0001
Empowerment	8.51	8.37–8.65	7.43	7.26–7.60	<0.0001
Appreciation, support & recognition	7.34	7.14–7.54	6.35	6.15–6.55	<0.0001
Receive regular payment	-0.33	-0.48 - -0.18	-0.75	-0.89 - -0.62	<0.0001
Perceived value of HSC meeting	7.07	6.77–7.38	4.15	3.78–4.51	<0.0001
Perceived responsibility of supervisor	8.63	8.37–8.88	5.85	5.49–6.21	<0.0001
Job satisfaction & attachment	7.08	6.93–7.23	5.99	5.82–6.16	<0.0001
**Behavioral Outcomes**					
Take initiative & problem solve	7.57	7.37–7.76	7.30	7.07–7.53	0.0878
Provide equitable services	6.70	6.30–7.10	4.99	4.58–5.40	<0.0001
*Evidence of teamwork*					
Colleagues hide information	1.75	1.69–1.80	1.49	1.43–1.56	<0.0001
Colleagues help each other	1.75	1.70–1.80	1.45	1.40–1.52	<0.0001
Colleagues help problem solve	1.44	1.37–1.50	1.33	1.26–1.39	0.0181
Frequency of interaction	8.74	8.62–8.87	8.35	8.22–8.48	<0.0001
Frequency of joint home visits	8.19	8.02–8.36	6.95	6.74–7.16	<0.0001
Frequency of meetings attended	9.66	9.63–9.82	8.47	8.27–8.67	<0.0001
*Evidence of supportive supervision*					
Fulfillment of supervisory duties	8.88	8.69–9.07	7.58	7.34–7.81	<0.0001
*Scenarios*					
Child gets sick, can't work	1.81	1.76–1.86	1.56	1.49–1.63	<0.0001
Busy month, need to prioritize home visits	1.88	1.84–1.92	1.86	1.82–1.91	0.5744
Complimentary feeding counsel, mother-in-law disagrees	1.54	1.48–1.60	1.16	1.11–1.22	<0.0001
Immunization counsel, mother refuses to listen	1.78	1.74–1.83	1.57	1.52–1.62	<0.0001
Stock-out of IFA tablets	1.00	0.96–1.03	0.96	0.93–1.00	0.1924
Pregnant mother in critical condition while away at relatives	1.95	1.93–1.97	1.87	1.84–1.91	0.0004

In particular, intervention FLHWs performed better on outcomes related to teamwork attitudes and behaviors, including the ‘Teamwork’ psychosocial measure (mean = 8.79 vs. 7.25); ‘Perceived value of the HSC meeting’, where the FLHWs came together for consultation (mean = 7.07 vs. 4.15); and both the ‘Frequency of joint home visits’ (mean = 8.19 vs. 6.95) and ‘Frequency of meetings attended’ (mean = 9.66 vs. 8.47).

The results show that the TBGI intervention positively impacted both motivational determinants, such as appreciation, support, and recognition (mean = 7.34 vs. 6.35), as well as motivational outcomes–job satisfaction and attachment (mean = 7.08 vs. 5.99). Of interest, intervention FLHWs demonstrated enhanced empowerment (mean = 8.51 vs. 7.43), which included questions related to self-efficacy to work as team and counsel women, particularly Dalit or Muslim women (minority groups). We also found a positive impact on the provision of equitable services (to Dalit women) among intervention FLHWs (mean = 6.70 vs. 4.99). Finally, intervention FLHWs were significantly more aware of their supervisors’ responsibilities (mean = 8.63 vs. 5.85) and more likely to report that their supervisors performed supervisory duties (mean = 8.88 vs. 7.58).

Compared to the FLHWs, the analysis for supervisors demonstrated fewer positive impacts of the intervention (Table 4). However, like with the FLHWs, intervention supervisors reported enhanced social cohesion and positive attitudes towards teamwork (mean = 8.37 vs. 5.29). We also found improvements on a few key measures of supervisory support, including ‘Encouraging FLHWs to solve a problem’ (mean = 1.85 vs. 1.59) and ‘Empowerment’—confidence in performing their supervisory duties of reviewing records, providing feedback, encouraging problem solving, and motivating the FLHWs (mean = 9.65 vs. 8.93). Finally, intervention supervisors were more likely to report that their group members (FLHWs) appreciated their help (mean = 1.90 vs. 1.80).

**Table 4:** Intervention effect on supervisors.

	Intervention (N = 52)		Control (N = 46)		P value
	Mean	CI	Mean	CI	
**Psychosocial Dimensions of Motivation**					
Teamwork	8.37	7.84–8.89	5.29	4.44–6.15	<0.0001
Empowerment	9.65	9.48–9.83	8.93	8.53–9.34	0.0010
*Appreciation*, *support & recognition*					
Receive regular payment	0.96	0 .60–1.33	0.91	0 .50–1.32	0.8590
Group members appreciate your help	1.90	1.82–1.99	1.63	1.45–1.81	0.0050
Family is proud of your work	1.90	1.82–1.99	1.80	1.69–1.92	0.1634
*Perceived value of HSC meeting*					
Find the HSC meeting pleasant	1.90	1.80–2.00	1.67	1.49–1.86	0.0272
Find the HSC meeting stressful	1.69	1.46–1.93	1.41	1.12–1.70	0.1345
Find the HSC meeting informative	1.79	1.63–1.95	1.46	1.19–1.73	0.0313
Perceived responsibility as a supervisor	7.96	7.51–8.41	7.45	6.87–8.03	0.1618
Job satisfaction & attachment	8.98	8.51–9.46	8.28	7.66–8.90	0.0725
**Behavioral Outcomes**					
*Evidence of supportive supervision*					
Encourage FLHWs to solve a problem	1.85	1.75–1.95	1.59	1.38–1.79	0.0196
Help FLHWs to solve a problem	1.79	1.65–1.93	1.70	1.51–1.88	0.4173
Tell FLHWs achieving targets is more important than teamwork	0.56	0.33–0.79	0.30	0.09–0.52	0.1123
Frequency of interaction	8.72	8.31–9.13	8.37	7.86–8.88	0.2785
*Scenarios*					
FLHW not doing work properly	1.60	1.42–1.77	1.50	1.28–1.72	0.4943
FLHW asks for advice & support	1.81	1.70–1.92	1.67	1.53–1.82	0.1322
Beneficiary targets falling for immunization	1.27	1.11–1.43	1.02	0 .92–1.12	0.0108

**Fig 3:**
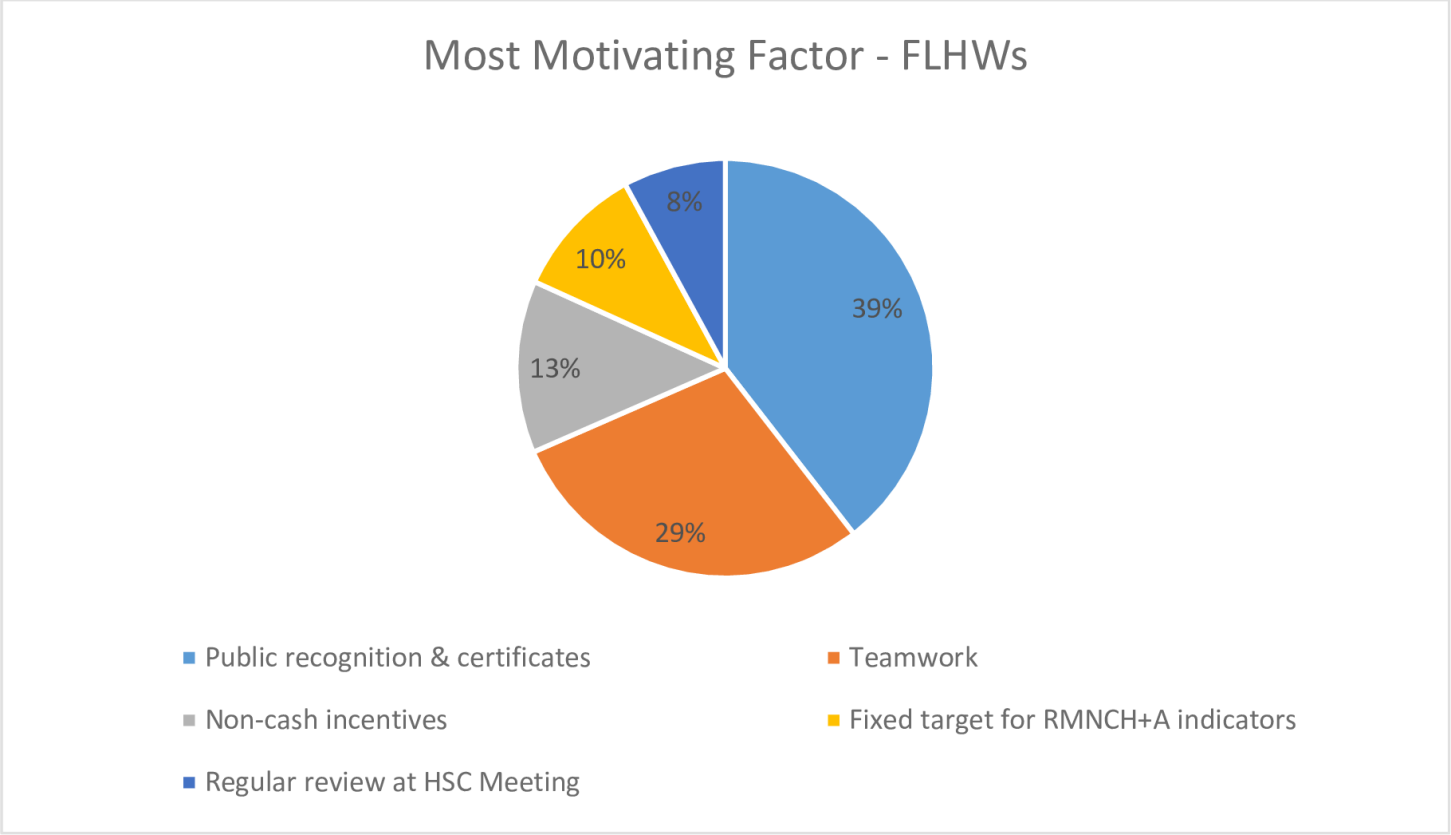
Most motivating factor for FLHWs.

**Fig 4:**
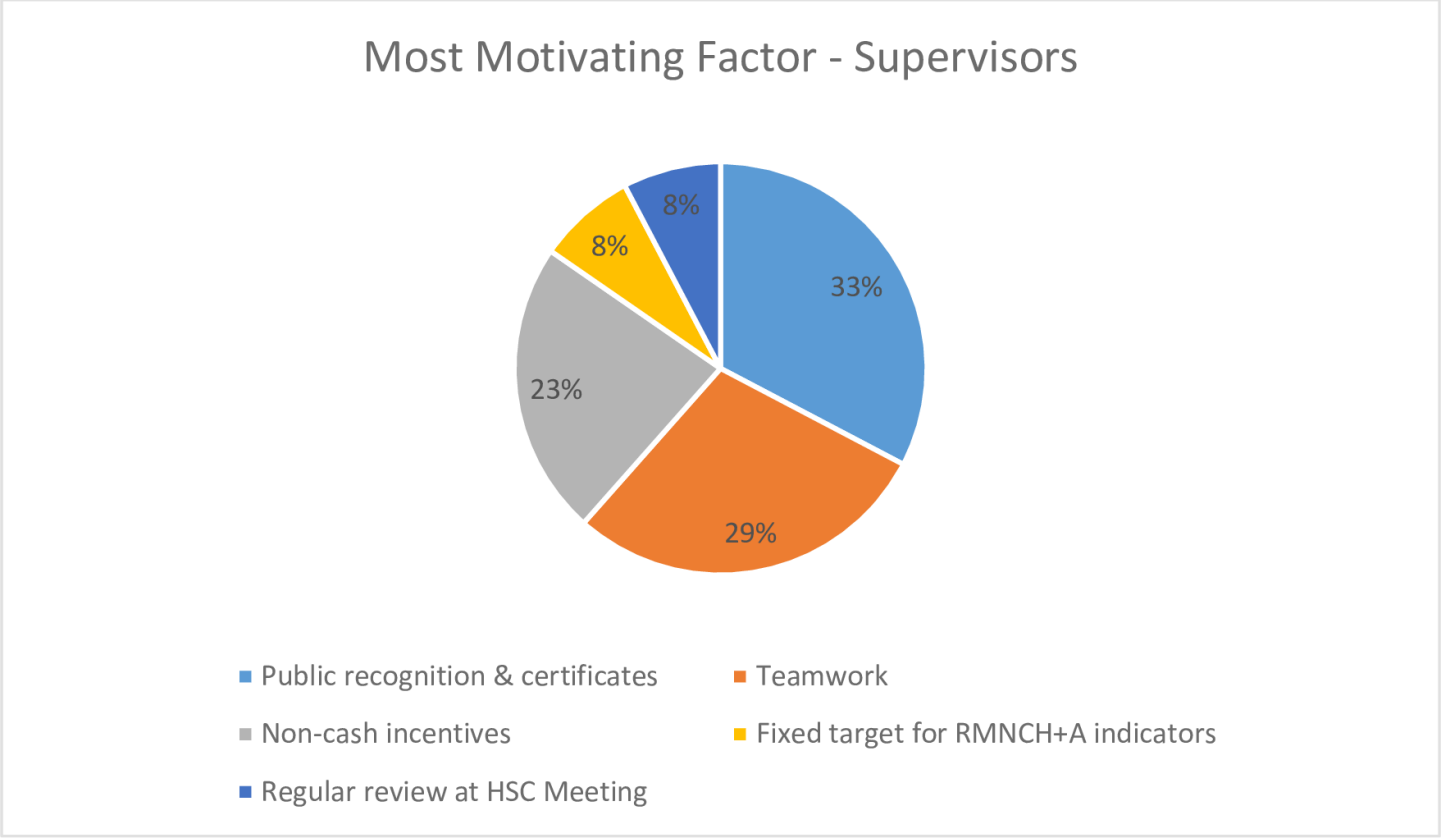
Most motivating factor for supervisors.

Regarding questions on the perceived value of the various intervention activities, both FLHWs and supervisors in the intervention groups identified ‘working together’ as the most important process that had made their job easier, 57.7% (CI 53.01–62.41) and 51.9% (37.88–65.97), respectively. Of note, the FLHWs and ANMs reported that they found public recognition, through the awarding of certificates by government officials at public meetings, to be the most important factor affecting their motivation [FLHWs: 39.5% (34.84–44.14), supervisors: 32.7% (CI 19.51–45.88)]. This was followed by reports of ‘teamwork’, 28.9% (24.66–33.29) for FLHWs and 28.9% (CI 16.11–41.58) for supervisors. See Figs 3 and 4.

As we were interested in the power of non-financial incentives, we asked several questions regarding this component specifically. The majority of the FLHWs and supervisors reported that they would continue to try and achieve the targets, even if the non-financial incentives were to stop, 96.9% (95.33–98.6) and 98.1% (94.22–100.00), respectively. When asked why, the leading motive cited was related to a belief that it was their duty and responsibility to do so, [FLHWs: 71% (66.71–75.34), supervisors: 76.9% (65.08–88.77)]; followed by working for a greater cause [FLHWs: 15.7% (CI 12.2–19.11), supervisors: 23.1% (11.23–34.92)]. When asked to explain in their own words the impact of the non-financial incentives on their families, 75% (70.88–79.12) of the FLHWs and 78.9% of the supervisors (67.37–90.33) reported that it made their family happy, and 34% (29.38–38.38) of the FLHWs and 26.9% (14.45–39.39) of the supervisors responded that it increased their family’s respect for their work.

## Discussion

We designed the TBGI intervention to address the work environment challenges faced by Bihar’s FLHWs, in a resource-constrained health system. CARE hypothesized that these outcomes could be achieved by supporting the FLHWs and supervisors to work as teams towards collective goals and by rewarding success through public recognition and non-financial incentives.

Our results support our hypothesis and indicate that the TBGI intervention is particularly well-suited for improving teamwork and supervisory support. In line with our hypothesis, the FLHWs who worked together towards collective goals and received rewards and recognition for their achievements were more motivated as compared to those FLHWs who did not have shared targets and incentives, as evidenced by the enhanced job satisfaction outcomes. Further, the intervention FLHWs demonstrated improved performance in the areas of teamwork and provision of equitable services. While we did not observe higher levels of job satisfaction among supervisors, there were improvements in a few key dimensions of motivation and supervisory performance outcomes. Our results also highlight TBGI’s power to impact motivational factors at the individual-level, including empowerment, family support, and intrinsic determinants, as well as its power to address challenges in the Indian health system.

As previously reported, Bihar’s FLHWs often lacked cohesion and accountability, had few interactions between them, and rarely sought support from their supervisors [10, 18, 19]. Given this, it is significant that TBGI led to increased information sharing, consultation, joint home visits, and monthly meeting attendance, as well as enhanced attitudes towards team members and confidence in working as a team. Other studies suggest that interventions that facilitate group identities, positive working relationships, and team climate, as well as reward groups rather than individuals, can improve motivation, enhance performance, and reduce turnover [8, 9, 26]. Our results demonstrate that TBGI is an effective model for advancing team climate and improving performance behaviors related to teamwork. These results are supported by similar findings in the previous RCT of TBGI [22]. TBGI may be particularly useful in contexts similar to India, where there are multiple cadres of FLHWs serving a single community.

The intervention FLHWs also reported increased self-efficacy to perform work duties, including working as a team and providing services, particularly to marginalized groups. This is important because evidence shows that self-efficacy contributes to motivation and job satisfaction [2, 11, 27]. Also of interest, the study found enhanced performance behaviors related to both teamwork and provision of equitable services. While further multivariable analysis would be necessary to fully explore the causal relationship between confidence and ability to perform work duties, our results signify the potential of TBGI to impact both.

We had expected to see a significant impact on intervention FLHWs’ behaviors related to problem solving and taking initiative, as the TBGI intervention actively promoted these behaviors; but we did not. This could be due to the inadequacy of the measure for this outcome (Cronbach’s alpha = 0.62). We did, however, observe significant differences for several of the scenarios, which aimed to assess these particular skills.

There is strong evidence that supervisory support is pivotal in improving health worker motivation and performance [9, 28]. Given this, as well as the number of studies from India [7, 10, 19, 27, 29] that call for enhanced supervisory support, the findings of this study are important and offer a model for addressing the gaps in supervisory support. Our results demonstrate that the FLHWs who regularly consulted and worked with their ANMs were significantly more aware of the support they could expect from their supervisors and reported improved supervisory behaviors, including enhanced assistance, checking of records, and provision of feedback.

While several items related to supervisory attitudes and performance among the ANMs lacked significance, the intervention ANMs were certainly more confident in their ability to perform their supervisory duties, which as noted earlier, evidence tells us that enhanced self-efficacy contributes to motivation and satisfaction. The intervention ANMs also reported greater appreciation from those they supervise. A study in Pakistan found that health workers who had supervisory duties were motivated, not only by the supervisory support they received, but also by the supervisory support they *provide* [28]. Our finding may suggest that the ANMs were positively impacted not only by the additional training and support they received as part of the intervention, but by the supervision they provide as well.

Regarding the intervention itself, we found that the FLHWs and supervisors found the public recognition ceremonies and certificates to be the most motivating component of the intervention, more so than the non-financial incentives. This finding corroborates other studies’ conclusions on the significant role recognition and appreciation play in health worker motivation [21, 28], including the previous TBGI RCT [22]; and reinforces recommendations to create opportunities for recognition as a means of motivating and supporting the development of FLHWs [7, 9, 27]. Participants did report that the incentives positively impacted their families’ respect and general happiness; and among FLHWs, we observed enhanced sense of appreciation, support, and recognition from community and family. These findings are important, as evidence suggests that family and community support and respect for one’s work can reinforce pride and the intrinsic motivation derived from one’s job [27, 28]. Intervention participants also reported they would work to achieve service targets, even if the non-financial incentives were to cease, citing as reasons: a sense of responsibility and belief in working for a greater cause. While this study cannot determine TBGI’s contribution to this altruism, key components of the intervention, such as the motivational pledge that included a commitment to ‘improve the health of our entire community, irrespective of caste, religion or geographic distance’, did focus on intrinsic motivation.

These observations are in line with other studies that identify the important roles that prestige, respect, family approval, confidence, professional satisfaction, and altruism play in influencing health worker motivation and performance [9, 11, 21, 28]. Including a study from India that found FLHWs were more motivated by individual- and community-level factors of public recognition, social responsibility, and self-efficacy than health system determinants, such as incentives and workloads, though the latter still mattered [27]. This paper does not seek to minimize the importance of compensation or financial incentives in motivating and retaining qualified health workers, as several studies have identified this as influential [7, 11, 27, 29]. As FLHWs serve as the backbone of many health systems–it is critical that they are properly compensated for their work. However, as argued in several other studies we cannot rely on financial compensation alone and need to further explore and leverage the beneficial impacts of non-financial determinants on health worker motivation and performance, particularly in countries with limited health budgets [7, 9, 13, 27, 29]. Therefore, this paper proposes that the TBGI model, which offers other forms of validation for one’s work, may be particularly useful in the contexts of rural India and in similar resource-constrained settings that have cadres of FLHWs without clear paths for career advancement or salary increases.

Finally, the findings from our study demonstrate the potential benefits of the TBGI model in addressing a couple of challenges within the Indian and other, similar health systems. A review of five remuneration models for community health workers, one of which was India’s incentive-based scheme for ASHAs, found that incentivized remuneration triggers a narrow focus on incentivized activities and limits attention given to broader community engagement [29]. This is supported by other studies’ findings that the incentive scheme in India prevented ASHAs from fulfilling other tasks, such as visiting and counseling people, and detracted from their capacity to act as broader health educators and advocates in their communities [19, 30]. TBGI’s model of rewards and recognition for a broader base of services with a heavy emphasis on home visits and counseling offers an approach for counter-balancing the current focus on government-incentivized services. Further, the TBGI model can motivate the FLHWs to perform their other, perhaps neglected, duties of counseling and health activism. This conclusion is supported by the findings of the earlier TBGI RCT, which found improvements in the frequency of FLHW-beneficiary interactions during critical times of care (final trimester of pregnancy and within 24 hours of delivery) and in the quality of advice provided by FLHWs during home visits, particularly for complimentary feeding and family planning [22]. The RCT also found that intervention FLHWs were more likely to counsel on a diverse range of maternal health topics, including non-incentivized topics such as advice on danger signs and saving money for delivery.

Another challenge the Indian health system faces that TBGI can help address is that while ASHAs have an intricate understanding of community health issues, as they live in the communities they serve, their insights often go untapped [29]. The hierarchical structure of India’s health system, in which health professionals rarely engage with or consult ASHAs, may result in missed opportunities to address the barriers and facilitators to community health service utilization. Since ASHAs have first-hand knowledge of these barriers, they could potentially be used more effectively to address issues such as the role of the mother-in-law and traditional practices in inhibiting healthy behavior and healthcare seeking. On this front, the TBGI model’s emphasis on facilitating teamwork, communication, and consultation among the FLHWs could help reduce the rigidness of the health system. Further, the monthly health sub-center meetings provide a platform for the ASHAs and AWWs to share the challenges they are facing with the ANMs, who can in turn communicate the challenges to the upper echelons of administration. Using the TBGI model as a mechanism for gathering information on what may or may not work on the ground and more purposefully funneling the information through the ANMs to the HSCs could prove an effective way to improve services.

## Limitations

During the conceptualization and design of the TBGI intervention, we gave greater attention to the motivation and performance factors related to the ASHAs and AWWs than to the supervisors. For example, the TBGI targets, and thus the associated rewards and recognition, were primarily related to job responsibilities of the ASHAs and AWWs rather than those of the supervisors, and many of the activities conducted during the HSC meetings focused on capacity building of the ASHAs and AWWs. Likewise, we first developed the questionnaire with the ASHAs and AWWs in mind and later adapted it for the ANMs. This focus may have resulted in lower performing measures and fewer significant results for the ANMs. Given the role supervisory support plays in FLHW motivation and performance, and thus a well-functioning health system, there is a need for more interventions designed to target supervisors specifically, as well as for further research to understand the supervisors’ motivational determinants and perspectives.

Since the motivational outcomes of interest in this study were not collected at baseline, we cannot estimate the change in the outcomes over time, and that limits our ability to estimate the true difference the intervention may have made. However, the randomized design, and the differences seen in these outcomes post-intervention, give us confidence that these differences are a result of exposure to the intervention. Further, although the population of ASHAs, AWWs, and ANMs per HSC is relatively stable, some individuals may have joined the workforce more recently and, therefore, had less exposure than others to the intervention, suggesting that the differences seen are a conservative estimate of the intervention effect.

We note that there were a few significant demographic differences in the two groups of FLHWs at the time of data collection, and since we restricted our analysis to bivariate associations, we did not take into account these differences. However, as we also note, although the intervention group had more FLHWs than the control group from either the general caste or scheduled caste/tribe, and who had been in their job for 5–10 years, the differences were relatively small, and we do not think it is likely that these differences would have a significant effect on the outcomes of interest.

Finally, as this study was conducted in a single district of Bihar, we cannot fully speak to its replicability. However, the Government of Bihar has since supported the scale-up of the intervention state-wide. Further research to determine the success of this scale-up would be beneficial in better understanding this intervention’s replicability.

## Conclusion

Team-Based Goals and Incentives offers a model for improving the work environment and enhancing health worker motivation and performance through a combination of joint goal setting, structured teamwork and recognition. The TBGI intervention had a significant impact on attitudes, self-efficacy, and associated behaviors of collaboration, consultation, and working together to achieve performance goals. TBGI also positively impacted factors related to supervisory support, including FLHWs’ understanding and perceptions of their supervisors and the supervisors’ confidence in fulfilling their duties. Finally, the TBGI model reinforces intrinsic motivation and covers areas of motivation not addressed through strictly incentive-based models. These finding are significant given health worker motivation has been recognized as essential to achieving national and global health goals and universal health coverage in low income countries.

Further, our findings contribute to the ongoing debate on the nature and importance of incentives and non-financial factors in influencing health worker motivation and performance. The TBGI intervention model may be particularly useful for programs operating within resource-constrained health settings and where multiple cadres of health workers—both paid professionals and auxiliary community health workers—need to work together to achieve the best results.

Although our sample size did not allow us to analyze differences in the response to the intervention by the two different cadres of FLHW (ASHAs and AWW), analysis of these differences might shed additional light on how best to bring together and support different types of health workers. Multivariable analysis might also further elucidate the relationship between the motivational factors and the performance outcomes. Further attention is also needed in designing and evaluating interventions specifically for FLHWs with supervisory duties.

## Supporting information

S1 Survey ANM survey tool. (PDF)

S2 Survey ASHA-AWW survey tool. (PDF)

S1 Data ASHA-AWW data and codebook. (XLSX)

S2 Data ANM data and codebook. (XLSX)
