# Supplementary material for: 'We pledge to improve the health of our entire community': Improving health worker motivation and performance in Bihar, India through teamwork, recognition, and non-financial incentives
Source: PLoS One. 2018 Aug 30;13(8):e0203265. doi: 10.1371/journal.pone.0203265 (PMC6117047; doi:10.1371/journal.pone.0203265)
Supplement: S1 Survey — (PDF) [file pone.0203265.s001.pdf]

TEAM BASED GOAL INCENTIVE (TBGI) SURVEY

UNDER BIHAR TECHNICAL SUPPORT PROJECT, CARE-INDIA

INTERVIEW SCHEDULE FOR ANM.

CONFIDENTIAL

(For TSU Project Purpose Only)

IDENTIFICATION

साक्षात्कारकर्ता के लिए निर्देश: हरेक भाग के शुरुआत में दिए गए भूरे रंग के बक्से में आपके लिए कुछ निर्देश दिए हुए हैं जो आपको पढ़ प्रश्न पूछने के पहले उतरदाता को पढ़ कर सुनाना है. आप सिर्फ उस भाग को पढ़े जिसमे निर्देश लिखा हुआ है न की उसके ऊपर दिए गए टाइटल को पढ़े. प्रत्येक सवाल पूछने के बाद दायी साइड में दिए गए संभव उतर को पढ़े. परन्तु अगर दायी साइड में अतिरिक्त निर्देश दिए हुए है तो कृप्या उसका पालन करे.

Instructions for enumerator: The grey boxes at the beginning of each survey section include a script for you to read to the interviewee before asking the questions…" "Only read the script indicted by "Script", the title and description of the section should NOT be read aloud." "After reading each question, read the possible response options in the right column, unless otherwise indicated with [Additional Instructions]"

A1. State:	Bihar
A2. District	Begusarai
A3. Community Development Block:	Bacchwara……………………………..1<br>Bhagwanpur…………………………..2<br>Birpur……………………………….....3<br>Chhaurahi……………………………..4<br>Navkothi………………………………5
A4. Health Sub Centre (Name)________________________	
A5. HSC category (circle one) :	Intervention ………………………….1<br>Control………………………………...2
A6. FLW category (circle one) :	AWW………………………………….1<br>ASHA………………………………….2
A7. Name of respondent (FLW)________________________	
A.8 .Place of interview:	AWC…………………………………….1<br>Home…………………………………….2<br>School…………………………………...3<br>Health sub Centre ……………………..4<br>Community Hall……………………….5<br>Other places…………………………….8
A.9 Results status	Completed.....……………………...……1<br>Refused after partial completion ……..2<br>Refused for interview…………………..3
A.10 Reason for refusal(for coded 2 and 3 in B1)____________	
A.11 Number of visits made__________________________	

	SPOT CHECKED BY	BACK CHECKED BY	EDITED BY	KEYED BY
Name	________________	________________	________________	________________
Date	________________	________________	________________	________________

Name of Investigator______________________ Signature of the Investigator___________________

Time of starting interview Hours --------------------------- Minutes ---------------------------

परिचय एवं सूचित सहमति INTRODUCTION AND INFORMED CONSENT

नमस्ते! मेरा नाम ____________________________ है ओर मै केयर इंडिया के लिए काम करता हूँ. इस सर्वे का उद्देश्य, केयर आई. एफ.एच. आई. परियोजना को यह समझने में मदद करना की उनके द्‌वारा फ्रंट लाइन वर्कर को मदद करने के लिए कार्यान्वित intervention प्रभावी रही हैं. आपके जवाब 'केयर' को बिहार में अपने काम को सुधारने में मूल्यवान मदद होगा. इसमें कोई भी उत्तर सही या गलत नहीं है. आपको उन प्रश्नों का उत्तर देने की जरुरत नहीं है जिसका उत्तर आप देना नहीं चाहती हैं. इस सर्वे के सारे प्रश्न गुप्त एवं नामरहित हैं और इसे आपके किसी भी साथी या सुपरवाइजर के साथ साझा नहीं किया जायेगा. अगर आपको किसी भी सवाल से असुविधा महसूस होती है तो आप हमें बताएँ हम उस सवाल को छोड़ अगले सवाल पर जायेंगे. क्या अब मै आपसे सवाल पूछना शुरु कर सकता हूँ? शुरू करने से पहले क्या आप सर्वेक्षण के बारे में कुछ मुझसे पूछना चाहती है?

[Script:] Thank you for speaking with me today. The purpose of this survey is to help the CARE India Integrated Family Health Initiative project understand if the interventions that have been implemented to help Frontline Health Workers have been effective. Your responses will be very valuable in helping CARE improve their work in Bihar. There are no right or wrong answers. You do not have to answer any questions you do not want to answer. All of the questions in this survey are completely confidential and anonymous and will not be shared with your co-workers or supervisor. If at any time you want to stop the interview, we can. If you are not comfortable answering a question, just let me know and we can skip it.

SURVEY FOR ANMs

Q. No.	QUESTIONS & FILTERS	CODING CATEGORIES
	**Background Characteristics** (पृष्ठभूमि)<br>**निर्देश: अब में आपसे, आपके बारे में कुछ सवाल करूँगा. मैं हरेक सवाल एवं उसके संभव उत्तर पढ़ कर सुनाऊंगा. इन हरेक सवाल के लिए आप मुझे वो जवाब दे जो आप पर लागू होता है.**<br>***[Script:]*** *First, I will ask you a few questions about yourself. I will read each question and then possible response options. For each of the questions tell me all answers that apply to you.*	
101	क्या आप उसी गाँव में रहते हैं जहाँ उप-स्वास्थ्य केंद्र है? Do you live in the same village where the sub-centre is located?	हाँ Yes.. 1<br>नहीं No.. 2
102	आप कितने दिनों से इस गाँव में रह रहीं हैं? Since how long have you lived in this village?	वर्षो ______________________________<br>Years
103	आप अपने केंद्र से कितनी दूर पर रहती हैं? How far from the sub-centre do you live?	दूरी कि. मी. में____________________<br>Distance in KM
104	आपकी वर्तमान आयु क्या है? What is your current age?	पूर्ण वर्ष<br>Completed years _______________
105	आपके धर्म क्या है? What is your religion?	हिन्दू Hindu……………………………………1<br>मुसलिम Muslim………………………………..2<br>क्रिस्चियन Christian……………………………..3<br>सिक्ख Sikh……………………………………..4<br>कोई धर्म नहीं No religion…………………………..5<br>अन्य Other _________________________________6<br>(स्पष्ट करें/ specify)
106	आप की जाति क्या हैं? What is your caste?	अनुसुचित जाति Schedule caste.................................1<br>अनुसुचित जनजाति Schedule tribe............................2<br>अन्य पिछड़ी जाति Other backward class...................3<br>सामान्य जाति General caste.......................................4
107	आपकी वैवाहिक स्थिति क्या है?What is your marital status?	वर्तमान में विवाहित Currently married.........................1<br>विधवा Widowed...2<br>तलाकशुदा Divorced..3<br>परित्यक्त Separated...4<br>अविवाहित Never married.....................................5
108	क्या आपने कभी कॉलेज में पढाई की है या कॉलेज स्तरीय पाठ्यक्रम में भाग लिया है या कोई डिप्लोमा लिया है? Have you attended college, or taken college level courses and received a diploma?	हाँ Yes.. 1<br>नहीं No.. 2
109	आपके उच्चतम शिक्षा कहाँ तक प्राप्त की है? What is your highest level of qualification?	उच्चतम शिक्षा स्तर<br>Highest level of education___________________________
110	आप इस पद पर कब से काम कर रही हैं? For how long have you been working in this position?	वर्ष Years___________________<br>महीना Months_______________
111	आप इस उप-केंद्र में कब से काम कर रही हैं? For how long have you been working in this sub-centre?	वर्ष Years___________________<br>महीना Months_______________
112	क्या आप गृह भ्रमण करती हैं? Do you do home visits?	हाँ Yes.. 1<br>नहीं No.. 2

Q. No.	QUESTIONS & FILTERS	CODING CATEGORIES
113.	आपने पिछले सप्ताह कितने गृह भ्रमण किया हैं? How many home visits did you do in the last week?	कुल गृह भ्रमण की संख्या<br>Total number Visit_______________________________
	**Teamwork: Social Cohesion (दल कार्य: सामाजिक एकजुटता)**<br>*[निर्देश]:* अब मैं आपसे, आपके साथी कार्यकर्ता के साथ आपके सम्बन्ध कैसे हैं और एक साथ टीम में काम करने के बारे में पूछूँगा. मैं पहले कथन को पढ़ूँगा. मेरे कथन पढ़ने के बाद आप बताएं की क्या आप उस कथन से "*पूरी तरह से सहमत, सहमत, असहमत और पूरी तरह से असहमत हैं*".<br>***[Script:]*** *I will now ask about your relationship with your co-workers and working together as a team. I will read a statement. After I read the statement, tell me whether you strongly agree, agree, disagree or strongly disagree.*	
114	आपको उनलोगों का साथ अच्छा लगता है जिनके साथ आप काम करती हैं. You enjoy the people you work with	पूरी तरह से सहमत Strongly agree....................................4<br>सहमत Agree...3<br>असहमत Disagree...2<br>पूरी तरह से असहमत Strongly disagree.............................1
115	आप जिन लोगो के साथ करते हैं वो आपके साथ इज्ज़त और सम्मान के साथ व्यवहार करते हैं The people you work with treat you with respect.	पूरी तरह से सहमत Strongly agree....................................4<br>सहमत Agree...3<br>असहमत Disagree...2<br>पूरी तरह से असहमत Strongly disagree.............................1
116	प्रायः उनलोगों के बीच मनमुटाव रहता है जिनके साथ आप काम करती हैं Often there is conflict among the people you work with.	पूरी तरह से सहमत Strongly agree....................................4<br>सहमत Agree...3<br>असहमत Disagree...2<br>पूरी तरह से असहमत Strongly disagree.............................1
117	सामान्य रूप से आप जिनके साथ काम करते हैं वो केवल अपने बारे में सोचते हैं In general, the people you work with only worry about themselves.	पूरी तरह से सहमत Strongly agree....................................4<br>सहमत Agree...3<br>असहमत Disagree...2<br>पूरी तरह से असहमत Strongly disagree.............................1
	**Perception about group (दल के बारे में आपकी अनुभूति)**<br>*[निर्देश:]* इस भाग में आपसे *आशा, आंगनवाड़ी के उस दल के बारे में पूछूँगा जिसका सुपरविजन (पर्यवेक्षण) आप करती हैं.* मैं पहले कथन को पढ़ूँगा. मेरे कथन पढ़ने के बाद आप बताएं की क्या आप उस कथन से "*पूरी तरह से सहमत, सहमत, असहमत और पूरी तरह से असहमत हैं*".<br>***[Script:]*** *I will now ask you a few questions about the group of ASHAs and AWWs you supervise. I will read a statement. After I read the statement, tell me whether you strongly agree, agree, disagree or strongly disagree.*	
118	समूह के सदस्य एक दुसरे की मदद करते हैं. The group members help each other	पूरी तरह से सहमत Strongly agree....................................4<br>सहमत Agree...3<br>असहमत Disagree...2<br>पूरी तरह से असहमत Strongly disagree.............................1
119	आप के विचार में, आप अपने समूह के सदस्यों पर भरोसा नहीं हैं की वो आपको कार्य क्षेत्र के बारे में सही जानकारी दें. You cannot count on them to give you accurate information about field reality**.**	पूरी तरह से सहमत Strongly agree....................................4<br>सहमत Agree...3<br>असहमत Disagree...2<br>पूरी तरह से असहमत Strongly disagree.............................1
120	आप के विचार में, आप के समूह के सदस्य दिए गए किसी भी कार्य को समय रहते पूरा कर लेगे. **If** given a particular task your group members will be able to accomplished it within an allotted time period	पूरी तरह से सहमत Strongly agree....................................4<br>सहमत Agree...3<br>असहमत Disagree...2<br>पूरी तरह से असहमत Strongly disagree.............................1
121	आपके के समूह के सदस्य एक दुसरे से सही और जरुरी जानकारियों छुपाते हैं. Your group members hide appropriate information from each other	पूरी तरह से सहमत Strongly agree....................................4<br>सहमत Agree...3<br>असहमत Disagree...2

Q. No.	QUESTIONS & FILTERS	CODING CATEGORIES
		पूरी तरह से असहमत Strongly disagree..............................1
122	आपके ग्रुप के कुछ सदस्य अपने कार्य के दायित्व को पूरा करने में उचित एवं पर्याप्त रूप से योगदान नहीं देते. Some members of your group do not contribute appropriately & adequately in fulfilling work duties.	पूरी तरह से सहमत Strongly agree.....................................4<br>सहमत Agree..3<br>असहमत Disagree...2<br>पूरी तरह से असहमत Strongly disagree..............................1
**Teamwork: Outcome expectations for teamwork (ग्रुपकार्य: ग्रुप कार्य से परिणाम की उम्मीद)**		
123	जब आशा और आंगनवाड़ी सेविका एक साथ काम करती है तो आप का काम आसान हो जाता है. When the ASHAs and AWWs work together as a team, your job is made easier.	पूरी तरह से सहमत Strongly agree.....................................4<br>सहमत Agree..3<br>असहमत Disagree...2<br>पूरी तरह से असहमत Strongly disagree..............................1
Empowerment: Self-efficacy & Confidence (सशक्तिकरण: आत्म दक्षता एवं आत्मविश्वास)<br>***[निर्देश]: इस भाग में, मैं आपसे पूछूँगा की आप को कितना विश्वास है की आप अलग-अलग परिस्थितियों में कोई काम कर सकती हैं. मैं पहले कथन को पढ़ूँगा. मेरे पढ़ने के बाद आप बताएं की "हाँ, आपको पूरा विश्वास है, हाँ, थोडा बहुत विश्वास है, नहीं, मुझे बहुत विश्वास नहीं है, नहीं, बिलकुल भी विश्वास नहीं हैं ".***<br>*[Script:] In this section, I will ask how confident you are that you can do something under different situations. I will read a statement. After I read the statement, tell me if you are completely confident, somewhat confident, not very confident or not at all confident in each situation.*		
124	आपको कितना भरोसा है की आप रिकॉर्ड और रिपोर्ट्स के सत्यता की समीक्षा/जाँच कर सकती हैं? How confident are you that you can review the records and reports of ASHA and AWW of your HSC for accuracy?	आपको पूरा विश्वास है ....4<br>Yes, completely confident I can.<br>हाँ, थोडा बहुत विश्वास है ....3<br>Yes, somewhat confident I can.<br>नहीं, मुझे बहुत विश्वास नहीं है<br>No, not very confident I can...2<br>नहीं, बिलकुल भी विश्वास नहीं हैं<br>No, not at all confident I can...1
125	आपको कितना भरोसा है की आप आशा/आंगनवाडी सेविका के प्रदर्शन पर प्रतिक्रिया (फीडबैक) दे सकती हैं? How confident are you that you can provide direct feedback to ASHA and AWW of your HSC about their performance?	आपको पूरा विश्वास है ....4<br>Yes, completely confident I can.<br>हाँ, थोडा बहुत विश्वास है ....3<br>Yes, somewhat confident I can.<br>नहीं, मुझे बहुत विश्वास नहीं है<br>No, not very confident I can...2<br>नहीं, बिलकुल भी विश्वास नहीं हैं<br>No, not at all confident I can...1
126	आपको कितना भरोसा है की आप अपने उप-स्वास्थ्य केंद्र के आशा/आंगनवाडी सेविका की मदद के लिए तकनीकी जानकारी एवं संसाधन प्रदान कर सकती हैं? How confident are you that you can provide technical resources or information to help ASHA and AWW of your HSC to do their job better?	आपको पूरा विश्वास है ....4<br>Yes, completely confident I can.<br>हाँ, थोडा बहुत विश्वास है ....3<br>Yes, somewhat confident I can.<br>नहीं, मुझे बहुत विश्वास नहीं है<br>No, not very confident I can...2<br>नहीं, बिलकुल भी विश्वास नहीं हैं<br>No, not at all confident I can...1
127	आपको कितना भरोसा है की आप आशा/आंगनवाडी सेविका का मनोबल बढ़ा सकती हैं? How confident are you that you can motivate ASHA and AWW of your HSC?	आपको पूरा विश्वास है ....4<br>Yes, completely confident I can.<br>हाँ, थोडा बहुत विश्वास है ....3<br>Yes, somewhat confident I can.<br>नहीं, मुझे बहुत विश्वास नहीं है<br>No, not very confident I can...2<br>नहीं, बिलकुल भी विश्वास नहीं हैं<br>No, not at all confident I can...1

Q. No.	QUESTIONS & FILTERS	CODING CATEGORIES
128	आपको कितना भरोसा है की आप अपने उप-स्वास्थ्य केंद्र के आशा/आंगनवाडी सेविका को समर्थ (सशक्त) बना सकती है?How confident are you that you can Empower ASHA and AWW of your HSC to solve problems on their own?	आपको पूरा विश्वास है ....4<br>Yes, completely confident I can.<br>हाँ, थोडा बहुत विश्वास है ....3<br>Yes, somewhat confident I can.<br>नहीं, मुझे बहुत विश्वास नहीं है<br>No, not very confident I can....2<br>नहीं, बिलकुल भी विश्वास नहीं हैं<br>No, not at all confident I can....1
**Job Satisfaction & Attachment (कार्य संतुष्टि एवं लगाव)**<br>निर्देश: इस भाग में, मै आप से जानना चाहता हूँ की आप अपने नौकरी एवं इस काम को करने के लिए मिलने वाले समर्थन और मान्यताओं के बारे में क्या महसूस करती हैं. मैं पहले कथन को पढ़ूँगा. मेरे कथन पढ़ने के बाद आप बताएं की क्या आप उस कथन से "*पूरी तरह से सहमत, सहमत, असहमत और पूरी तरह से असहमत हैं*". याद रखें की आप के द्वारा दिए गए उत्तर गोपनीय है और इसे किसी से भी साझा (शेयर) नहीं किया जायेगा.<br>*Now I will ask how you feel about your job and the support and recognition you receive for doing your job. I will read a statement. After I read each statement, tell me if you strongly agree, agree, disagree or strongly disagree. Remember, your answers are confidential and will not be shared with anyone.*		
संतुष्टि **Satisfaction**:		
129	मोटा-मोटी आप अपनी नौकरी से खुश हैं Overall, you are satisfied with your work.	पूरी तरह से सहमत Strongly agree....4<br>सहमत Agree....3<br>असहमत Disagree....2<br>पूरी तरह से असहमत Strongly disagree....1
**Overall Motivation (पूर्ण प्रोत्साहन)**		
130	आपको उस काम को करने में आनंद आता है जिसमे उत्तरदायित्व ज्यादा होता है. You enjoy work that requires great responsibilities	पूरी तरह से सहमत Strongly agree....4<br>सहमत Agree....3<br>असहमत Disagree....2<br>पूरी तरह से असहमत Strongly disagree....1
131	जब भी कोई मुश्किल काम आता है आप जल्दी हार मान जाती हैं. When faced with a difficult task you tend to give up quickly.	पूरी तरह से सहमत Strongly agree....4<br>सहमत Agree....3<br>असहमत Disagree....2<br>पूरी तरह से असहमत Strongly disagree....1
**Sense of doing valuable work (अहम काम करने की भावना)**		
132	आप कुछ और करना चाहेंगी अगर आपको दूसरा काम मिल जाता है. You would do something else if you could get another job.	पूरी तरह से सहमत Strongly agree....4<br>सहमत Agree....3<br>असहमत Disagree....2<br>पूरी तरह से असहमत Strongly disagree....1
133	आप अपने उप-स्वास्थ्य केंद्र के मिशन में विश्वास करती हैं. You believe in the mission of your health sub-centre.	पूरी तरह से सहमत Strongly agree....4<br>सहमत Agree....3<br>असहमत Disagree....2<br>पूरी तरह से असहमत Strongly disagree....1
134	आप ये काम सिर्फ पैसे के लिए करती हैं. You only do this job for money.	पूरी तरह से सहमत Strongly agree....4<br>सहमत Agree....3<br>असहमत Disagree....2<br>पूरी तरह से असहमत Strongly disagree....1
135	आप उन अवसरों से संतुष्ट हैं, जिसमें आपको आपकी क्षमताओं का उपयोग करने का मौका	पूरी तरह से सहमत Strongly agree....4<br>सहमत Agree....3<br>असहमत Disagree....2

Q. No.	QUESTIONS & FILTERS	CODING CATEGORIES
	मिलता हैं. You are satisfied with the opportunity to use your abilities in this job.	पूरी तरह से असहमत Strongly disagree……………………………1
**Appreciation and Recognition (सराहना एवं मान्यता)**		
136	आपको अपने काम का उचित एवं नियमित वेतन मिलता है. You receive regular and reliable payment for your work.	पूरी तरह से सहमत Strongly agree……………………………….4<br>सहमत Agree……………………………………………………………3<br>असहमत Disagree………………………………………………….2<br>पूरी तरह से असहमत Strongly disagree……………………………1
137	ज्यादातर ग्रुप के सदस्य आपकी मदद की सराहना करते हैं. Most of your group members you meet with really appreciate your help.	पूरी तरह से सहमत Strongly agree……………………………….4<br>सहमत Agree……………………………………………………………3<br>असहमत Disagree………………………………………………….2<br>पूरी तरह से असहमत Strongly disagree……………………………1
138	आपके परिवार को आपके काम पर नाज है. Your family is proud of the work that you do.	पूरी तरह से सहमत Strongly agree……………………………….4<br>सहमत Agree……………………………………………………………3<br>असहमत Disagree………………………………………………….2<br>पूरी तरह से असहमत Strongly disagree……………………………1
**व्यवहार संबंधी परिणाम: नेतृत्व भूमिका लेना (Behavioural Outcomes: Taking up a leadership role)**<br>*[निर्देश]: अब मैं आपसे जानना चाहते हूँ की आप उन आशा, आंगनवाड़ी सेविका से अकसर कितनी बार बातचीत करती हैं, जिन्हें आप सुपरवाइज़ करती हैं.*<br>***[Script:]*** *Now I would like to know how often you interact with the ASHA and AWW you supervise.*		
139	आप अकसर कितनी बार अपने ग्रुप के सदस्यों से बातचीत करती हैं. How often do you interact with your team members?	प्रति दिनEvery day …………………………………….1<br>सप्ताह में एक बार Once in a week……………………2<br>पंद्रह दिन में एक बार Once in a fortnight…………….3<br>महीने में एक बार Once in a month……………………4<br>शायद ही कभी Rarely…………………………………….5<br>कभी नहीं Never…………………………………………...6
140	पिछली बार आपने कब किसी आशा/ आंगनवाड़ी सेविका से ऑफिस मीटिंग के अलावा बातचीत की थी. When was the last time you interacted with AWW/ASHA in your HSC other than the Official meeting?	पिछले महीने Last month……………………………….1<br>पिछले पंद्रह दिनों में Last fortnight……………………2<br>पिछले सप्ताह Last week……………………………….3<br>इसी सप्ताह This week………………………………….4<br>कभी नहीं Never………………………………………….5
141	पिछली बार आशा, आंगनवाड़ी सेविका के साथ आपने गृह भ्रमण कब किया था . When was the last time you did a joint home visit with ASHA/AWW from your HSC?	पिछले महीने Last month……………………………….1<br>पिछले पंद्रह दिनों में Last fortnight……………………2<br>पिछले सप्ताह Last week……………………………….3<br>इसी सप्ताह This week………………………………….4<br>कभी नहीं Never………………………………………….5
[निर्देश]: आप आशा और आंगनवाड़ी सेविका जिन्हें आप सुपरवाइज़ करते हैं, के साथ कैसे काम करते हैं? ***मैं प्रत्येक कथन को पढ़ूँगा. कथन को पढ़ने के बाद, आप बताएं की आप इनमें से हरेक कार्य को हमेशा, प्रायः, शायद ही कभी, कभी भी नहीं करती है.***<br>***[Script:]*** *Now I would like to ask a few questions about how you work with the ASHAs and AWWs you supervise. I will read a statement. After I read the statement, tell me whether you think you do each action Always, Often, Rarely or Never.*		
142	जब आपके ग्रुप सदस्यों में से किसी को कोई समस्या होती है तो आप उन्हें प्रोत्साहित करती है की वो समाधान ढूँढने के लिए कोशिश करती रहे. When any of your group members encounters a problem, you encourage them to keep trying to find a solution.	हमेशा Always…………………………………………………………4<br>प्राय: Often………………………………………………………………3<br>शायद ही कभी Rarely……………………………………………….2<br>कभी ही नहीं Never……………………………………………………1

Q. No.	QUESTIONS & FILTERS	CODING CATEGORIES
143	जब आपके ग्रुप सदस्य में से कोई, किसी समस्या का सामना करती है तो आप उनके लिए समस्या का समाधान करती हैं. When one of your group members encounters a problem, you ~~solve it for them~~help them solve it.	हमेशा Always.....4<br>प्राय: Often.....3<br>शायद ही कभी Rarely.....2<br>कभी ही नहीं Never.....1
144	आप अपने ग्रुप के सदस्यों को बोलते है की ग्रुप में काम करने के बजाय टारगेट को हासिल करना ज्यादा महत्वपूर्ण हैं. You tell your group it is more important for them to accomplish their target than it is for them to work as a team.	हमेशा Always.....4<br>प्राय: Often.....3<br>शायद ही कभी Rarely.....2<br>कभी ही नहीं Never.....1
**उप-स्वास्थ्य केंद्र के बैठक एवं प्रोत्साहन का कथित मूल्य: Perceived value of the HSC meetings**<br>**निर्देश: अब में आपसे पूछना चाहूँगा की आप उप-स्वास्थ्य केंद्र की बैठक के बारे में क्या महसूस करती हैं? *मैं एक कथन को पढ़ूँगा. कथन को पढ़ने के बाद, आप बताएं की क्या आप नीचे दिए गए एहसास को हमेशा, प्रायः, शायद ही कभी, या कभी नहीं महसूस करती है.***<br>***[Script:]*** *Now I would like to ask a few questions about how you feel about the HSC meeting. I will read a statement. After I read the statement, tell me whether you feel that way Always, Often, Rarely or Never.*		
145	आप उप-स्वास्थ्य केंद्र के मीटिंग को सुखद पाती हैं. You find HSC meeting very pleasant	हमेशा Always.....4<br>प्राय: Often.....3<br>शायद ही कभी Rarely.....2<br>कभी ही नहीं Never.....1
146	आप उप-स्वास्थ्य केंद्र की मीटिंग को तनाव पूर्ण पाती है. You find HSC meeting very stressful.	हमेशा Always.....4<br>प्राय: Often.....3<br>शायद ही कभी Rarely.....2<br>कभी ही नहीं Never.....1
147	आप उप-स्वास्थ्य केंद्र की मीटिंग को सूचनाप्रद पाती है. You find HSC meeting very informative.	हमेशा Always.....4<br>प्राय: Often.....3<br>शायद ही कभी Rarely.....2<br>कभी ही नहीं Never.....1

**ANM Leadership**<br>***[निर्देश]***: *क्या आप ये महसूस करती हैं की नीचे दिए गए काम को करने की जिम्मेवारी आपकी है? मेरे प्रत्येक कथन के पढ़ने के बाद आप बतायें की क्या आप उस कथन से "पूरी तरह से सहमत, सहमत, असहमत और पूरी तरह से असहमत हैं".*<br>***[Script]:*** *I would like to know if you feel that as an ANM it is your responsibility to do a particular thing. After I read each statement, tell me whether you Strongly Agree, Agree, Disagree or Strongly Disagree it is your responsibility.*		
148	आशा और आंगनवाडी सेविका को टारगेट पूरा करने के फायदे की व्याख्या करना. It is your responsibility to Explain the benefits of achieving their targets to ASHA and AWW	पूरी तरह से सहमत Strongly agree.....4<br>सहमत Agree.....3<br>असहमत Disagree.....2<br>पूरी तरह से असहमत Strongly disagree.....1
149	अपने ग्रुप के आशा और आंगनवाडी सेविका के प्रदर्शन के लिए अपने को जिम्मेवार मानना. It is your responsibility to Be accountable for the performance of the ASHA and AWW in your HSC	पूरी तरह से सहमत Strongly agree.....4<br>सहमत Agree.....3<br>असहमत Disagree.....2<br>पूरी तरह से असहमत Strongly disagree.....1
150	अपने उप-स्वास्थ्य केंद्र के आशा और आंगनवाडी सेविका को नेतृत्व प्रदान करना. It is your responsibility to Provide leadership to ASHA and AWW in your HSC	पूरी तरह से सहमत Strongly agree.....4<br>सहमत Agree.....3<br>असहमत Disagree.....2

		पूरी तरह से असहमत Strongly disagree............................1
151	अपने उप-स्वास्थ्य केंद्र के आशा और आंगनवाडी सेविका को स्पष्ट मिशन बताना. It is your responsibility to Communicate a clear vision and mission to the ASHA and AWW of your HSC	पूरी तरह से सहमत Strongly agree...................................4<br>सहमत Agree..3<br>असहमत Disagree...2<br>पूरी तरह से असहमत Strongly disagree............................1
152	ग्रुप के सदस्यों के बीच सब को साथ लेकर चलने के व्यवहार (समावेशता/समग्रता) को प्रोत्साहित करती है. It is your responsibility to Foster inclusiveness among the ASHA and AWW in your HSC	पूरी तरह से सहमत Strongly agree...................................4<br>सहमत Agree..3<br>असहमत Disagree...2<br>पूरी तरह से असहमत Strongly disagree............................1
153	वैसे वातावरण का निर्माण करना जिसमे की आपके क्षेत्र के आशा और आंगनवाडी सेविका अपनी राय व्यक्त कर सकते हैं. It is your responsibility to Create an environment where differences of opinion can be voiced by ASHA and AWW in your area	पूरी तरह से सहमत Strongly agree...................................4<br>सहमत Agree..3<br>असहमत Disagree...2<br>पूरी तरह से असहमत Strongly disagree............................1
154	अपने उप-स्वास्थ्य केंद्र के आशा और आंगनवाडी सेविका के बीच के मनमुटाव का समाधान करना. It is your responsibility to Resolve conflict among the ASHA and AWW of your HSC	पूरी तरह से सहमत Strongly agree...................................4<br>सहमत Agree..3<br>असहमत Disagree...2<br>पूरी तरह से असहमत Strongly disagree............................1
155	आशा और आंगनवाडी सेविका को स्पष्ट दायित्व देना और उनको इस बात की अनुमति देना की वो अपने तरीके से काम को पूरा करे. It is your responsibility to Provide clear responsibilities to ASHA and AWW and allow them to decide how to accomplish them.	पूरी तरह से सहमत Strongly agree...................................4<br>सहमत Agree..3<br>असहमत Disagree...2<br>पूरी तरह से असहमत Strongly disagree............................1
156	अपने सुविधा के अनुसार अपने ग्रुप के सदस्यों को निर्देश देना. It is your responsibility to Instruct and order your group as per your convenience.	पूरी तरह से सहमत Strongly agree...................................4<br>सहमत Agree..3<br>असहमत Disagree...2<br>पूरी तरह से असहमत Strongly disagree............................1
[ ] अब में आप से कुछ सवाल आपके द्वारा किये जाने वाले नियमित कार्य से सम्बंधित है, के बारे में पूछना चाहूँगा<br>***[Script:]*** *Now I would like to ask you few question related to you regular work activities*		
157	आप अपने ग्रुप के आशा और आंगनवाडी सेविका के रिकॉर्ड और रिपोर्ट्स के सत्यता की समीक्षा/जाँच करती हैं? Do you review the records and reports of ASHA and AWW in your group for accuracy in the at Health Sub-center meeting?	हाँ Yes.. 1<br>नहीं No.. 2
158	आप गृह भ्रमण के दौरान अपने उप-स्वास्थ्य केंद्र के आशा और आंगनवाडी सेविका का कार्यो का अवलोकन करती है? Do you Observe the ASHA and AWW in your HSC during their home visits?	हाँ Yes.. 1<br>नहीं No.. 2
159	आप अपने उप-स्वास्थ्य केंद्र के आशा और आंगनवाडी सेविका के प्रदर्शन पर प्रतिक्रिया (फीडबैक) देती हैं. Do you regularly Provide the ASHA and AWW in your HSC with direct feedback about their performance?	हाँ Yes.. 1<br>नहीं No.. 2
160	अपने उप-स्वास्थ्य केंद्र के आशा/आंगनवाडी सेविका के काम को बेहतर बनाने के लिए उन्हें जरूरी तकनीकी जानकारी एवं संसाधन प्रदान करना? Do you Provide the ASHA and AWW in your HSC with	हाँ Yes.. 1<br>नहीं No.. 2

	technical resources or information to help them do their job better?	
**Scenarios for Behavioral Outcomes**<br>***निर्देश: अब मैं आपके सामने अलग-अलग सिनेरियो और कई अलग-अलग विकल्पों को रखूँगा. प्रत्येक कथन के पढ़ने के बाद आप अपने शब्दों में बतायें की आप क्या सोचती है की उन अलग-अलग परिस्थितियों में आप क्या करेगी?***<br>*[Script:] Now I will present you with several different scenarios and several different options for what you would do in each situation. After I read each statement, tell me in your own words what you think you would do in each situation.*		
161	कल्पना कीजिये की उप-स्वास्थ्य केंद्र के आशा/आंगनवाडी सेविका अपना काम सही तरीके से नहीं कर रही है तो ऐसी स्थिति में आप क्या करती हैं? Imagine that an ASHA/ AWW in your sub-center is not doing her work properly. What do you do?	**अतिरिक्त निर्देश (जोर से ना पढ़े):** कृप्या विकल्पों को ना पढ़े? आशा आंगनवाडी सेविका को खुली प्रतिक्रिया देने दें, उसके बाद उस विकल्प पर गोला करें जो आशा/आंगनवाड़ी सेविका के दिए गए जवाब से सबसे ज्यादा मिलता जुलता है. इसका सिर्फ एक ही जवाब हो सकता है. **[Additional Instructions (do not read aloud):** DO NOT READ OPTIONS. Allow for free response from the ASHA/AWW, then circle the response option that most closely matches what the ASHA/AWW says. There can only be ONE response.]<br><br>उसकी व्यवहार को प्रखंडस्तरीय पदाधिकारी को रिपोर्ट करेंगी. Report her behavior to the Block officials……………………………1<br><br>आप उससे ये जानने के लिए बात करेंगी की काम में कोई समस्या तो नहीं हो रही है और उसे मदद का प्रस्ताव देंगी. Talk to her to see if she is facing challenges at work and offer to help……………………………………………………………………………2<br><br>उसे आप अतिरिक्त प्रशिक्षण देंगी. Provide her with additional training……………………………………………………………………3<br>आप उनके गृह भ्रमण के दौरान उनका मार्गदर्शन एवं मदद करेगी Accompany her on her home visits to provide guidance and support……………………………………………………………………4<br><br>दुसरे आशा/आंगनवाडी सेविका जो की अपने क्षेत्र में अच्छा कर रही है के साथ में टीम बनाकर एक सप्ताह के लिए काम करने, मार्गदर्शन एवं मदद करने के लिए बोलेगी. Ask another ASHA/AWW who is doing well to partner with her for a week or two and provide support and guidance……………………………………………………………………5<br><br>अन्य Other ____8<br><br>(स्पष्ट करें/ specify)
162	कल्पना करे कि, एक नवजात की माँ ने आशा/आंगनवाडी सेविका के द्वारा टीकाकरण के महत्व पर दिए गए परामर्श को मानने से इंकार कर दिया, उसके बाद आशा/आंगनवाडी सेविका आपके पास सलाह / मदद के लिए आती हैं तो आप किस तरह की मदद प्रदान करेगी? Imagine an ASHA/AWW comes to you for advice after she was turned away when she visited home of a newborn to counsel the mother on the importance of immunization. ASHA/AWW has come to you asking for help. . How do you support her?	**अतिरिक्त निर्देश (जोर से ना पढ़े):** कृप्या विकल्पों को ना पढ़े? आशा आंगनवाडी सेविका को खुली प्रतिक्रिया देने दें, उसके बाद उस विकल्प पर गोला करें जो आशा/आंगनवाड़ी सेविका के दिए गए जवाब से सबसे ज्यादा मिलता जुलता है. इसका सिर्फ एक ही जवाब हो सकता है. **[Additional Instructions (do not read aloud):** DO NOT READ OPTIONS. Allow for free response from the ASHA/AWW, then circle the response option that most closely matches what the ASHA/AWW says. There can only be ONE response.]<br><br>आप आशा/आंगनवाडी सेविका को बोलेगी की आप ने अपना काम कर दिया अब आप कुछ और नहीं कर सकती You tell the

		ASHA/AWW that she did her job, nothing else can be done………………………………………………………………………………………1<br>आप आशा/आंगनवाडी सेविका को उस घर का पुनः भ्रमण करने को बोलेगी. Advise the ASHA/AWW to visit that home again……………………………………………………………………………………..2<br>आप आशा/आंगनवाडी सेविका को सलाह देगी की उसके पति और सास से बात करे Advise the ASHA/AWW to talk to the husband and mother-in-law………………………………………………………………..3<br>आप उन्हें ये बोलेंगी की अगले गृह भ्रमण के दौरान उनके साथ होंगी और उस महिला से बात एवं मदद करेंगी. Offer to accompany the ASHA/AWW on her next home visit to talk to the woman and provide support…………………………………......................4<br><br>अन्य Other ____8<br><br>(स्पष्ट करें/ specify)
163	मान लीजिये की टीकाकरण सत्र के दौरान, आप ये महसूस करती हैं की लाभार्थियों की संख्या पिछले महीने की तुलना में कम रही है. आप क्या करेंगी? Suppose during Regular Immunization (RI) sessions, you notice that the number of beneficiaries are decreasing as compared to past month for service uptake; what would you do?	**अतिरिक्त निर्देश (जोर से ना पढ़े):** कृप्या विकल्पों को ना पढ़े? आशा आंगनवाडी सेविका को खुली प्रतिक्रिया देने दें, उसके बाद उस विकल्प पर गोला करें जो आशा/आंगनवाड़ी सेविका के दिए गए जवाब से सबसे ज्यादा मिलता जुलता है. इसका सिर्फ एक ही जवाब हो सकता है. **[Additional Instructions (do not read aloud):** DO NOT READ OPTIONS. Allow for free response from the ASHA/AWW, then circle the response option that most closely matches what the ASHA/AWW says. There can only be ONE response.]<br><br>आप डुय लिस्ट की जाँच करेंगे की सारे लाभार्थी ठीक से सूचीबद्ध है की नहीं? Have the ASHA and AWW Check the due list to see if all beneficiaries are listed properly………………………………………………………………………………1<br>आशा और आंगनवाडी सेविका को उनके काम में लापरवाही के लिए डांटते हैं. Scold ASHA and AWW for their negligence in work……………………………………………………………………………………….2<br><br>आशा और आंगनवाड़ी सेविका से पूछते हैं की क्षेत्र में क्या हो रहा है? Ask the ASHA and AWWs about what is happening on the ground...3<br>आशा और आंगनवाड़ी सेविका से पूछते हैं की परिस्थिति में सुधार लाने के लिए क्या करना चाहिए? Ask the ASHA and AWW what they think needs to be done to improve the situation…………………….4<br>आशा और आंगनवाड़ी को समस्या का समाधान ढूँढने के लिए प्रोत्साहित करते हैं. Encourage the ASHA and AWWs to solve the issue Work with the ASHA and AWW to solve the issue…………………5<br><br>लाभार्थियों के उपस्थिति में अंतर होता रहता है इसलिए हमें कुछ विशेष करने की जरुरत नहीं है. Differences in beneficiaries occur-there’s no need to do anything specific……………………….6<br><br>अन्य Other ____8

		(स्पष्ट करें/ specify)

इस भाग के सवाल केवल उन आशा एवं आंगनवाडी सेविकायों के लिए है जो की टी. बी. जी. आई. के इंटरवेंशन के अंतर्गत आते हैं. The following section contains questions for those ASHAs and AWWs who received the TBGI INTERVENTION ONLY**.**

Q. No.	QUESTIONS & FILTERS	CODING CATEGORIES
	**टी. बी. जी. आई. का कथित मूल्य (**Perceived Value of the TBGI Intervention**)**<br>**निर्देश: अब मैं आपसे केयर इंडिया इंटरवेंशन के उस पक्ष के बारे में पूछना चाहूँगा जिससे आपका काम को आसान हो गया है और आपको काम करने के लिए प्रेरित करता है.** ***[Script:]*** *I would now like to ask you a few questions about what aspects of the CARE intervention has made your work easier and has most motivated you.*	
164	इनमें में से कौन सी प्रक्रिया ने आपके काम को आसान बना दिया है. Which is the most important process that has made your work easier?	विभिन्न सूचक के लिए निर्धारित टारगेट.............................. ☐<br>Fixed target for various indicators.<br><br>एक साथ काम करना.. ☐<br>Working together.<br><br>नियमित मासिक बैठक.. ☐<br>Regular monthly meeting.<br><br>उपलब्धियों की नियमित समीक्षा.. ☐<br>Regular review of achievement.
165	इनमें में से कौन सी प्रक्रिया ने आपके काम को आसान बना दिया है. Which is the **SECOND** most important process that has made your work easier?	विभिन्न सूचक के लिए निर्धारित टारगेट.............................. ☐<br>Fixed target for various indicators.<br><br>एक साथ काम करना.. ☐<br>Working together.<br><br>नियमित मासिक बैठक.. ☐<br>Regular monthly meeting.<br><br>उपलब्धियों की नियमित समीक्षा.. ☐<br>Regular review of achievement.
166	इसमें से कौन से फैक्टर (घटक) ने आपके ज्यादा काम करने की इच्छा को प्रभावित किया है. Which is the most important factor that has affected your motivation to work?	विभिन्न सूचक के लिए नियत टारगेट................................. ☐<br>Fixed target for various indicators.<br><br>एक साथ टीम के रूप में काम करना.................................. ☐<br>Team work.<br><br>गैर-मौद्रिक प्रोत्साहन.. ☐<br>Non-cash incentives.<br><br>सरकार के वरीय पदाधिकारी द्वारा दिए गए सर्टिफिकेट...... ☐<br>Certificates given by the senior government officials.<br><br>उप-स्वास्थ्य केंद्र के मासिक बैठक में काम की नियमित समीक्षा<br>Regular review of work at HSC meeting................................ ☐

167	इसमें से कौन से फैक्टर (घटक) ने आपके ज्यादा काम करने की इच्छा को प्रभावित किया है. Which is the **SECOND** most important factor that has affected your motivation to work?	विभिन्न सूचक के लिए नियत टारगेट................................ ☐<br>Fixed target for various indicators.<br><br>एक साथ टीम के रूप में काम करना.................................. ☐<br>Team work.<br><br>गैर-मौद्रिक प्रोत्साहन.. ☐<br>Non-cash incentives.<br><br>सरकार के वरीय पदाधिकारी द्वारा दिए गए सर्टिफिकेट...... ☐<br>Certificates given by the senior government officials.<br><br>उप-स्वास्थ्य केंद्र के मासिक बैठक में काम की नियमित समीक्षा<br>Regular review of work at HSC meeting................................ ☐
**Perceived Value of Non-Cash Incentives**<br>अब मैं आपसे नॉन-कैश (गैर-नकदी) प्रोत्साहन से सम्बंधित कुछ प्रश्न करना चाहूँगा.<br>***[Script:]*** *Now I would like to ask few questions regarding the non- cash incentives.*		
168	क्या आप जानते हैं की आपको प्रोत्साहन क्यों मिल रहा है? Do you know why are you getting the non-cash incentive? Please tell me in your own words why you think you are getting the non-cash incentive.	**[अतिरिक्त निर्देश (जोर से ना पढ़े)]:** कृप्या विकल्पों को ना पढ़े? आशा आंगनवाडी सेविका को खुली प्रतिक्रिया देने दें, उसके बाद उस विकल्प पर गोला करें जो आशा/आंगनवाड़ी सेविका के दिए गए जवाब से सबसे ज्यादा मिलता जुलता है. इसका सिर्फ एक ही जवाब हो सकता है. **[Additional Instructions (do not read aloud):** DO NOT READ OPTIONS. Allow for free response from the ASHA/AWW, then circle the response option that most closely matches what the ASHA/AWW says. There can only be ONE response.]<br><br>अच्छा काम करने के लिए To work well.....................................A<br><br>सारे लाभार्थियों तक पहुँचने के लिए To get reach of all the beneficiaries..B<br><br>बैठक को उचित तरीके से करने के लिए To conduct the meeting in proper manner..C<br><br>ग्रुप के सदस्यों अच्छा कम कर सके उसमे मदद करने के लिए To help the team member to work well.......................................D<br><br>समय पर टारगेट को पूरा करने के लिए. To achieve the target on time...E<br><br>अन्य Other ____X<br><br>(स्पष्ट करें/ specify)
169	क्या आप नॉन-कैश (गैर-नकदी) प्रोत्साहन से संतुष्ट हैं? Are you satisfied with the non-cash incentives:	हाँ Yes... 1<br>नहीं No... 2
170	आप नॉन-कैश (गैर-नकदी) प्रोत्साहन का उपयोग कैसे करती हैं? Please tell me in your own Word how do you utilize the Non-cash incentives:<br><br>.	**अतिरिक्त निर्देश (जोर से ना पढ़े):** कृप्या विकल्पों को ना पढ़े? आशा आंगनवाडी सेविका को खुली प्रतिक्रिया देने दें, उसके बाद उस विकल्प पर गोला करें जो आशा/आंगनवाड़ी सेविका के

		दिए गए जवाब से सबसे ज्यादा मिलता जुलता है. इसका सिर्फ एक ही जवाब हो सकता है. **[Additional Instructions (do not read aloud):** DO NOT READ OPTIONS. Allow for free response from the ASHA/AWW, then circle the response option that most closely matches what the ASHA/AWW says. There can only be ONE response.]<br><br>घर के कामों के लिए For household purpose..............................A<br><br>बेटी के शादी के लिए For daughters marriage.............................B<br><br>किसी और को उपहार देने के लिए As a gift to someone............C<br><br>किस काम का नहीं No use...D<br><br>अन्य Other ____X<br><br>(स्पष्ट करें/ specify)
171	अगर मान लीजिये कि किसी कारणवश प्रोत्साहन अचानक से बंद हो जाये, तो क्या आप फिर भी टारगेट को समय पर पूरा करने की कोशिश करेंगी?<br>Let us assume that due to some reason, non-cash incentive would stop all of sudden will you still try to achieve the target on time?	हाँ Yes.. 1<br>नहीं No... 2
172	अगर हाँ, तो बिना किसी प्रोत्साहन के टारगेट को पूरा करने के लिए आप क्यों अतिरिक्त मेहनत करेंगी.<br>If yes, without any incentive why would you put in extra effort to still achieve the target on time? Please tell me in your own words why you would still try to achieve the target on time.	**अतिरिक्त निर्देश (जोर से ना पढ़े):** कृप्या विकल्पों को ना पढ़े? आशा आंगनवाडी सेविका को खुली प्रतिक्रिया देने दें, उसके बाद उस विकल्प पर गोला करें जो आशा/आंगनवाड़ी सेविका के दिए गए जवाब से सबसे ज्यादा मिलता जुलता है. इसका सिर्फ एक ही जवाब हो सकता है. **[Additional Instructions (do not read aloud):** DO NOT READ OPTIONS. Allow for free response from the ASHA/AWW, then circle the response option that most closely matches what the ASHA/AWW says. There can only be ONE response.]<br><br>हमलोग बेहतर कारण के लिए काम रहे है................................A<br>We are working for better cause.<br><br>यह मेरा कर्तव/ दायित्तव हैं ....B<br>This is my duty/ responsibilities.<br>प्रोत्साहन मेरे लिए कोई मायने नहीं रखता...............................C<br>Incentive does not matter for me.<br>समूह कार्य के लिए...D<br>Team work.<br><br>अन्य Other ____X<br><br>(स्पष्ट करें/ specify)
173	नॉन-कैश (गैर-नकदी) प्रोत्साहन आपके परिवार वाले को इस बात के लिए सहमत करने में मदद के रहा हैं की आप और ज्यादा काम करे<br><br>Non-cash incentive is helping you to convince your family to allow you to work more?	पूरी तरह से सहमत Strongly agree.....................................4<br>सहमत Agree..3<br>असहमत Disagree..2<br>पूरी तरह से असहमत Strongly disagree.............................1

174	आपके परिवार में प्रोत्साहन मुख्य रूप से किसको प्रभावित करता है? Who does the non-cash incentive impact the most in your family?	बच्चे Children……………………………………………………………1<br>पति Husband……………………………………………………………2<br>घर के अन्य सदस्य other family member……………….......3
175	प्रोत्साहन ने किस तरह से आपके परिवारवालो को प्रभावित किया है? Please tell me in your own words what kind of impacts has the non-cash incentive had on your family members?<br><br>.	**अतिरिक्त निर्देश (जोर से ना पढ़े):** कृप्या विकल्पों को ना पढ़े? आशा आंगनवाडी सेविका को खुली प्रतिक्रिया देने दें, उसके बाद उस विकल्प पर गोला करें जो आशा/आंगनवाड़ी सेविका के दिए गए जवाब से सबसे ज्यादा मिलता जुलता है. इसका सिर्फ एक ही जवाब हो सकता है. **[Additional Instructions (do not read aloud):** DO NOT READ OPTIONS. Allow for free response from the ASHA/AWW, then circle the response option that most closely matches what the ASHA/AWW says. There can only be ONE response.]<br><br>ज्यादा आदर मिलता है क्योंकि नॉन-कैश प्रोत्साहन पैसे से ज्यादा असरदार है. Getting more respect because it works more than money…………………………………………………………………………A<br><br>यह आपके परिवार के सदस्यों के चेहरे पर ख़ुशी लाती है. It brings smile to the face of family member……………………………B<br><br>परिवारवालो से घर का काम काज ख़त्म करने में ज्यादा मदद मिलती है Getting more help from family member to finish household chore ……………………………………………………………C<br><br>मेरे काम का आदर/मूल्य बढ़ गया है. Respect/value has increased for my work……………………………………………………D<br><br>अन्य Other ____X<br><br>(स्पष्ट करें/ specify)
176	नॉन-कैश (गैर-नकदी) प्रोत्साहन ने आपके परिवार का आपके और आपके काम के प्रति दृष्टिकोण को बदल दिया है. Non-cash incentives have changed the perception of your family towards you and your work.	पूरी तरह से सहमत Strongly agree………………………………4<br>सहमत Agree……………………………………………………………3<br>असहमत Disagree……………………………………………………..2<br>पूरी तरह से असहमत Strongly disagree……………………….1
177	अगर आप सहमत है तो कृप्या व्याख्या करें, कैसे? If agree, could you explain, How?	**अतिरिक्त निर्देश (जोर से ना पढ़े):** कृप्या विकल्पों को ना पढ़े? आशा आंगनवाडी सेविका को खुली प्रतिक्रिया देने दें, उसके बाद उस विकल्प पर गोला करें जो आशा/आंगनवाड़ी सेविका के दिए गए जवाब से सबसे ज्यादा मिलता जुलता है. इसका सिर्फ एक ही जवाब हो सकता है. **[Additional Instructions (do not read aloud):** DO NOT READ OPTIONS. Allow for free response from the ASHA/AWW, then circle the response option that most closely matches what the ASHA/AWW says. There can only be ONE response.]<br><br>____________________<br>____________________<br>____________________<br>____________________<br>____________________
