# Supplementary material for: 'We pledge to improve the health of our entire community': Improving health worker motivation and performance in Bihar, India through teamwork, recognition, and non-financial incentives
Source: PLoS One. 2018 Aug 30;13(8):e0203265. doi: 10.1371/journal.pone.0203265 (PMC6117047; doi:10.1371/journal.pone.0203265)
Supplement: S2 Survey — (PDF) [file pone.0203265.s002.pdf]

TEAM BASED GOAL INCENTIVE (TBGI) SURVEY

UNDER BIHAR TECHNICAL SUPPORT PROJECT, CARE-INDIA
INTERVIEW SCHEDULE FOR ASHA/AWW.

CONFIDENTIAL

(For TSU Project Purpose Only)

IDENTIFICATION

साक्षात्कारकर्ता के लिए निर्देश: हरेक भाग के शुरुआत में दिए गए भूरे रंग के बक्से में आपके लिए कुछ निर्देश दिए हुए हैं जो आपको पढ़ प्रश्न पूछने के पहले उतरदाता को पढ़ कर सुनाना है. आप सिर्फ उस भाग को पढ़े जिसमे निर्देश लिखा हुआ है न की उसके ऊपर दिए गए टाइटल को पढ़े. प्रत्येक सवाल पूछने के बाद दायी साइड में दिए गए संभव उतर को पढ़े. परन्तु अगर दायी साइड में अतिरिक्त निर्देश दिए हुए है तो कृप्या उसका पालन करे.

Instructions for enumerator: The grey boxes at the beginning of each survey section include a script for you to read to the interviewee before asking the questions…" "Only read the script indicted by "Script", the title and description of the section should NOT be read aloud." "After reading each question, read the possible response options in the right column, unless otherwise indicated with [Additional Instructions]"

A1. State:	Bihar
A2. District	Begusarai
A3. Community Development Block:	Bacchwara……………………………1<br>Bhagwanpur…………………………2<br>Birpur……………………………...3<br>Chhaurahi……………………………4<br>Navkothi……………………………..5
A4. Health Sub Centre (Name)____	
A5. HSC category (circle one) :	Intervention …………………………1<br>Control………………………………..2
A6. FLW category (circle one) :	AWW…………………………………1<br>ASHA…………………………………2
A7. Name of respondent (FLW)____	
A.8 .Place of interview:	AWC……………………………………1<br>Home……………………………………2<br>School…………………………………...3<br>Health sub Centre ……………………..4<br>Community Hall……………………….5<br>Other places…………………………….8
A.9 Results status	Completed.....…………………...……1<br>Refused after partial completion ……..2<br>Refused for interview…………………..3
A.10 Reason for refusal(for coded 2 and 3 in B1)____________	
A.11 Number of visits made_________________________________	

	SPOT CHECKED BY	BACK CHECKED BY	EDITED BY	KEYED BY
Name	________________	________________	________________	________________
Date	________________	________________	________________	________________

Name of Investigator______________________ Signature of the Investigator___________________

Time of starting interview Hours --------------------------- Minutes ---------------------------

परिचय एवं सूचित सहमति INTRODUCTION AND INFORMED CONSENT

नमस्ते! मेरा नाम ____________________________ है ओर मै केयर इंडिया के लिए काम करता हूँ. इस सर्वे का उद्देश्य, केयर आई. एफ.एच. आई. परियोजना को यह समझने में मदद करना की उनके द्वारा फ्रंट लाइन वर्कर को मदद करने के लिए कार्यान्वित intervention प्रभावी रही हैं. आपके जवाब 'केयर' को बिहार में अपने काम को सुधारने में मूल्यवान मदद होगा. इसमें कोई भी उत्तर सही या गलत नहीं है. आपको उन प्रश्नों का उत्तर देने की जरुरत नहीं है जिसका उत्तर आप देना नहीं चाहती हैं. इस सर्वे के सारे प्रश्न गुप्त एवं नामरहित हैं और इसे आपके किसी भी साथी या सुपरवाइजर के साथ साझा नहीं किया जायेगा. अगर आपको किसी भी सवाल से असुविधा महसूस होती है तो आप हमें बताएँ हम उस सवाल को छोड़ अगले सवाल पर जायेंगे. क्या अब मै आपसे सवाल पूछना शुरु कर सकता हूँ? शुरू करने से पहले क्या आप सर्वेक्षण के बारे में कुछ मुझसे पूछना चाहती है?

[Script:] Thank you for speaking with me today. The purpose of this survey is to help the CARE India Integrated Family Health Initiative project understand if the interventions that have been implemented to help Frontline Health Workers have been effective. Your responses will be very valuable in helping CARE improve their work in Bihar. There are no right or wrong answers. You do not have to answer any questions you do not want to answer. All of the questions in this survey are completely confidential and anonymous and will not be shared with your co-workers or supervisor. If at any time you want to stop the interview, we can. If you are not comfortable answering a question, just let me know and we can skip it.

SURVEY FOR ASHAs/AWWs

Q. No.	QUESTIONS & FILTERS	CODING CATEGORIES
**Background Characteristics (पृष्ठभूमि)**<br>***निर्देश: अब में आपसे, आपके बारे में कुछ सवाल करूँगा. मैं हरेक सवाल एवं उसके संभव उत्तर पढ़ कर सुनाऊंगा. इन हरेक सवाल के लिए आप मुझे वो जवाब दे जो आप पर लागू होता है.***<br>***[Script:]*** *First, I will ask you a few questions about yourself. I will read each question and then possible response options. For each of the questions tell me all answers that apply to you.*		
101	क्या आप उसी गाँव में रहते हैं जहाँ उप-स्वास्थ्य केंद्र है? Do you live in the same village where the sub-center is located?	हाँ Yes ……………………………………………………….1<br>नहीं No……………………………………………………..2
102	आप कितने दिनों से इस गाँव में रह रहीं हैं? Since how long have you lived in this village?	वर्षों Years ____________________________________
103	आप अपने केंद्र से कितनी दूर रहती हैं? How far from the sub-center do you live?	पूर्ण वर्ष Completed years_________________________
104	आपके धर्म क्या है? What is your religion?	हिन्दू Hindu ……………………………………………..1<br>मुसलिम Muslim ………………………………………..2<br>क्रिस्चियन Christian ……………………………………3<br>सिक्ख Sikh ……………………………………………...4<br>कोई धर्म नहीं No religion………………………………5<br>अन्य Other____________________________________8<br>(स्पष्ट करें/Specify)
105	What is your caste? आप की जाति क्या हैं?	अनुसुचित जाति Schedule caste ………………………1<br>अनुसुचित जनजाति Schedule tribe……………………..2<br>अन्य पिछड़ी जाति Other backward class …………….3<br>सामान्य जाति General caste……………………………4<br>अन्य Other ____________________________________8<br>(स्पष्ट करें/Specify)
106	आपकी वैवाहिक स्थिति क्या है?What is your marital status?	वर्तमान में विवाहित Currently married ……………….1<br>विधवा Widowed ………………………………………..2<br>तलाकशुदा Divorced ……………………………………3<br>परित्यक्त Separated ……………………………………4<br>अविवाहित Never married ………………………………5
107	क्या आपने कभी कॉलेज में पढाई की है या कॉलेज स्तरीय पाठ्यक्रम में भाग लिया है या कोई डिप्लोमा लिया है? Have you attended college, or taken college level courses and received a diploma?	हाँ Yes ……………………………………………………….1<br>नहीं No……………………………………………………..2
108	आपके उच्चतम शिक्षा कहाँ तक प्राप्त की है? What is your highest level of qualification?	उच्चतम शिक्षा स्तर Highest level of education________
109	आप इस पद पर कब से काम कर रही हैं? For how long have you been working in this position?	वर्ष Years________________________________<br>महीना Months______________________________
110	आप इस उप-केंद्र में कब से काम कर रही हैं? For how long have you been working in this sub-center?	वर्ष Years______________________________<br>महीना Months____________________________

Q. No.	QUESTIONS & FILTERS	CODING CATEGORIES
111	आपकी ड्यूटी क्या है? What is your area(s) of responsibility?<br><br>**[Additional Instructions (do not read aloud):** DO NOT READ OPTIONS. Allow for free response from the ASHA/AWW, then circle all of the responses options that most closely match what the ASHA/AWW says. There may be multiple responses.]	**[आशा की जिम्मेदारियाँ ASHA area of responsibilities.]**<br><br>गर्भवती महिलायों का पंजीकरण pregnancy registration……………………………………………….A<br><br>सांस्थानिक प्रसव, ए न सी और पी न सी देखभाम सुनिश्चित करना. To ensure institutional delivery, ANC and PNC care………………………………………………………B<br><br>माता और बच्चों का टीकाकरण सुनिश्चित करना To ensure the immunization of mother and child…………………….C<br><br>सिर्फ और सिर्फ स्तनपान सुनिश्चित करना To ensure exclusive breast feeding …………………………………………..D<br><br>गृह भ्रमण Home visit ……………………......................E<br><br>**[आंगनवाडी कार्यकर्ता की जिम्मेदारियाँ AWW area of responsibilities.]**<br><br>अनौपचारिक शाला पूर्व शिक्षा non-formal Pre-school education………………………………………………..F<br><br>पोषक क्षेत्र का सर्व Survey of catchment area……………G<br><br>बच्चों का वज़न और ग्रोथ चार्ट तैयार करना To weigh children and prepare growth chart…………………….H<br><br>स्वस्थ और पोषण की शिक्षा और परामर्श Education and counselling in health and nutrition………………………I<br><br>गृह भ्रमण Home visit………………………….................J<br><br>किशोरियों को स्वास्थ्य और पोषण के बारे में परामर्श देना To counsel adolescent girls on nutrition and health……..K
112	क्या आप गृह भ्रमण करती हैं? Do you do home visits?	हाँ Yes ……………………………………………1<br>नहीं No……………………………………………2
113	आपने पिछले सप्ताह कितने गृह भ्रमण किया हैं? How many home visits did you do in the last week	कुल गृह भ्रमण की संख्या Total number Visit _________
114	आपने पिछले महीने किस तरह की सेवाएँ प्रदान की है? What services did you provide in your last home visit?	**[Additional Instructions (do not read aloud):** DO NOT READ OPTIONS. Allow for free response from the ASHA/AWW, then circle all of the responses options that most closely match what the ASHA/AWW says. There may be multiple responses.]<br><br>गर्भावस्था के समय ए न सी जांच के महत्व के बारे में बतलाना. Counsel about the importance of getting check-ups during pregnancy (ANC)………………………………………..A

Q. No.	QUESTIONS & FILTERS	CODING CATEGORIES
		गर्भावस्था के दौरान खतरे के लक्षण के बारे में बतलाना. Counsel about danger signs during pregnancy and childbirth ……………………………………………………B<br><br>प्रसव के लिए तैयारी के बारे में बतलाना Counsel about planning for delivery (such as how she will get to the facility, who will take care of her other children, and who will accompany her to the facility)……………………..C<br><br>एच आई वी की जांच के बारे में परामर्श Counsel about getting tested for HIV……………………………………………..D<br><br>बच्चे के जन्म के बाद सिर्फ और सिर्फ स्तनपान के बारे में परामर्श Counsel about exclusive breastfeeding once the baby is born………………………………………………...E<br><br>बच्चे के जन्म के बाद गर्भाधान में विलम्ब या वर्जित करने के नियोजन सम्बन्धी परामर्श Counsel about planning for contraception to avoid or delay another pregnancy after the baby is born……………………………………………………F<br><br>नाल की स्वच्छता अभ्यास के बारे में परामर्श Counsel about clean cord care practice ……………………………………..G<br><br>६-११ साल तक के उम्र के बच्चों को उनके उम्र के हिसाब से पूरक आहार के मात्रा के बारे में परामर्श Counsel about appropriate quantity of complementary feeding between 6-11 month of their age……………………………………..H<br><br>पूर्ण टीकाकरण के बारे में परामर्श Counsel about complete immunization……………………………………………..I<br><br>उच्च खतरे वाले नवजात के पहचान, उसकी देखभाल और रेफरल Identification of high risk neonates, their care and referral……………………………………………………..J<br><br>सेपसिस की पहचान, देखभाल और रेफरल Sepsis identification, care and referral…………………………………………..K
**Teamwork: Social Cohesion (दल कार्य: सामाजिक एकजुटता)**<br>निर्देश: अब मैं आपसे, आपके साथी कार्यकर्ता के साथ आपके सम्बन्ध कैसे हैं और एक साथ टीम में काम करने के बारे में पूछूँगा. मैं पहले कथन को पढ़ूँगा. मेरे कथन पढ़ने के बाद आप बताएं की क्या आप उस कथन से "*पूरी तरह से सहमत, सहमत, असहमत और पूरी तरह से असहमत हैं*".<br>***[Script:]*** *I will now ask about your relationship with your co-workers and working together as a team. I will read a statement. After I read the statement, tell me whether you strongly agree, agree, disagree or strongly disagree.*		
115	आपको उन लोगों का साथ अच्छा लगता है जिनके साथ आप काम करती हैं. You enjoy the people you work with	पूरी तरह से सहमत Strongly agree……………………………..4<br>सहमत Agree…………………………………………………………3<br>असहमत Disagree………………………………………………..2<br>पूरी तरह से असहमत Strongly disagree………………………1
116	आप जिन लोगों के साथ काम करते हैं वो आपके साथ इज्ज़त और सम्मान के साथ व्यवहार करते हैं	पूरी तरह से सहमत Strongly agree……………………………..4<br>सहमत Agree…………………………………………………………3<br>असहमत Disagree………………………………………………..2

Q. No.	QUESTIONS & FILTERS	CODING CATEGORIES
	The people you work with treat you with respect.	पूरी तरह से असहमत Strongly disagree……………………….1
117	प्रायः उन लोगों के बीच मनमुटाव रहता है जिनके साथ आप काम करती हैं Often there is conflict among the people you work with.	पूरी तरह से सहमत Strongly agree……………………………4<br>सहमत Agree…………………………………………………3<br>असहमत Disagree…………………………………………2<br>पूरी तरह से असहमत Strongly disagree……………………….1
118	सामान्य रूप से आप जिनके साथ काम करते हैं वो केवल अपने बारे में सोचते हैं In general, the people you work with only worry about themselves.	पूरी तरह से सहमत Strongly agree……………………………4<br>सहमत Agree…………………………………………………3<br>असहमत Disagree…………………………………………2<br>पूरी तरह से असहमत Strongly disagree……………………….1
119	आप जिन लोगों के साथ काम करते हैं उन में से अधिकांश लोगों पर आप विश्वास करते हैं You can trust the majority of people you work with.	पूरी तरह से सहमत Strongly agree……………………………4<br>सहमत Agree…………………………………………………3<br>असहमत Disagree…………………………………………2<br>पूरी तरह से असहमत Strongly disagree……………………….1
**Teamwork: Outcome expectations for teamwork (ग्रुपकार्य: ग्रुप कार्य से परिणाम की उम्मीद)**		
120	एक साथ काम करने से आप का काम आसान हो जाता है. By working as a team, your job is made easier.	पूरी तरह से सहमत Strongly agree……………………………4<br>सहमत Agree…………………………………………………3<br>असहमत Disagree…………………………………………2<br>पूरी तरह से असहमत Strongly disagree……………………….1
121	मान लीजिये की आप अपने रिश्तेदार के घर गई हैं जो की आपके कार्यक्षेत्र से काफी दूर है और उसी समय आपको एक ऐसी महिला का फ़ोन आता है जिसका की प्रसव होने वाला है और नाजुक अवस्था में है. आपको विश्वास है की आपकी कोई भी साथी उस महिला का दौरा करेगी अगर आप उन्हें दौरा करने को कहें. Suppose you have gone to visit your relative which is quite far from your work area and during that time you receive a call from a pregnant women who is about to deliver a baby and is in critical situation. Do you believe any of your co-workers would visit the woman for you if you asked them to?	पूरी तरह से सहमत Strongly agree……………………………4<br>सहमत Agree…………………………………………………3<br>असहमत Disagree…………………………………………2<br>पूरी तरह से असहमत Strongly disagree……………………….1
**Empowerment: Self-efficacy & Confidence (सशक्तिकरण: आत्म दक्षता एवं आत्मविश्वास)**<br>निर्देश: इस भाग में, मैं आपसे पूछूँगा की आप को कितना विश्वास है की आप अलग-अलग परिस्थितियों में कोई काम कर सकती हैं. मैं पहले कथन को पढ़ूँगा. मेरे पढ़ने के बाद आप बताएं की *"हाँ, आपको पूरा विश्वास है, हाँ, थोडा बहुत विश्वास है, नहीं, मुझे बहुत विश्वास नहीं है, नहीं, बिलकुल भी विश्वास नहीं हैं".*<br>***[Script:]*** *In this section, I will ask how confident you are that you can do something under different situations. I will read a statement. After I read the statement, tell me if you are completely confident, somewhat confident, not very confident or not at all confident in each situation.*		
122	आपको कितना यकीन है की आप उप-स्वास्थ्य केंद्र की मीटिंग में अपनी राय व्यक्त कर सकती हैं. How confident are you that you can express your opinion at a health sub-center meeting?	आपको पूरा विश्वास है ……………………………………………4<br>Yes, completely confident I can.<br>हाँ, थोडा बहुत विश्वास है ……………………………………3<br>Yes, somewhat confident I can.<br>नहीं, मुझे बहुत विश्वास नहीं है<br>No, not very confident I can……………………………………2<br>नहीं, बिलकुल भी विश्वास नहीं हैं<br>No, not at all confident I can……………………………………1

Q. No.	QUESTIONS & FILTERS	CODING CATEGORIES
123	आपको कितना यकीन है की आप अपने ए. न. एम से मदद मांग सकती हैं How confident are you that you can ask your ANM for help?	आपको पूरा विश्वास है ....4<br>Yes, completely confident I can.<br>हाँ, थोडा बहुत विश्वास है ....3<br>Yes, somewhat confident I can.<br>नहीं, मुझे बहुत विश्वास नहीं है<br>No, not very confident I can....2<br>नहीं, बिलकुल भी विश्वास नहीं हैं<br>No, not at all confident I can....1
124	आपको कितना यकीन है की आप अपने साथी कार्यकर्ता से मदद मांग सकती हैं. How confident are you that you can ask your co-workers for help?	आपको पूरा विश्वास है ....4<br>Yes, completely confident I can.<br>हाँ, थोडा बहुत विश्वास है ....3<br>Yes, somewhat confident I can.<br>नहीं, मुझे बहुत विश्वास नहीं है<br>No, not very confident I can....2<br>नहीं, बिलकुल भी विश्वास नहीं हैं<br>No, not at all confident I can....1
125	आपको कितना यकीन है की आप 6 महीने के उम्र के बच्चों वाली उन हरेक माँ, जिसमे की दलित एवं मुस्लिम महिला भी शामिल हैं, को पूरक आहार देने के लिए राज़ी कर सकती हैं. How confident are you that you can persuade every woman you visit, including a Dalit or Muslim woman, with a 6 month old child to begin complementary feeding practices?	आपको पूरा विश्वास है ....4<br>Yes, completely confident I can.<br>हाँ, थोडा बहुत विश्वास है ....3<br>Yes, somewhat confident I can.<br>नहीं, मुझे बहुत विश्वास नहीं है<br>No, not very confident I can....2<br>नहीं, बिलकुल भी विश्वास नहीं हैं<br>No, not at all confident I can....1
126	आपको कितना यकीन है की आप उन हरेक महिला को जिनके घर आप जाते हो, जिसमे की दलित एवं मुस्लिम महिला भी शामिल हैं, को देरी से गर्भवती होने के फायदे के बारे में बता कर देरी से गर्भवती होने के लिए राज़ी करवा सकती हैं. How confident are you that you can persuade every woman you visit, including a Dalit or Muslim woman, of the benefits of delaying a pregnancy?	आपको पूरा विश्वास है ....4<br>Yes, completely confident I can.<br>हाँ, थोडा बहुत विश्वास है ....3<br>Yes, somewhat confident I can.<br>नहीं, मुझे बहुत विश्वास नहीं है<br>No, not very confident I can....2<br>नहीं, बिलकुल भी विश्वास नहीं हैं<br>No, not at all confident I can....1
127	अगर कोई महिला देर से बच्चा चाहती है तो आपको कितना यकीन है की आप उन हरेक महिला जिसमे की दलित एवं मुस्लिम महिला भी शामिल हैं, को परिवार नियोजन तरीकों के उपयोग के बारे में राज़ी कर सकती हैं. If a woman wants to delay a pregnancy, how confident are you that you can persuade that woman, including a Dalit or Muslim woman, to use a family planning method?	आपको पूरा विश्वास है ....4<br>Yes, completely confident I can.<br>हाँ, थोडा बहुत विश्वास है ....3<br>Yes, somewhat confident I can.<br>नहीं, मुझे बहुत विश्वास नहीं है<br>No, not very confident I can....2<br>नहीं, बिलकुल भी विश्वास नहीं हैं<br>No, not at all confident I can....1
128	अगर एक महिला, और बच्चा नहीं चाहती है तो आपको कितना यकीन है की आप उस महिला को लम्बे समय के लिए परिवार नियोजन के तरीकों (नसबंदी) को अपनाने के लिए राज़ी कर सकती हैं. If a woman does not want any more children, how confident are you that you can persuade her to use a long-term family planning method (or sterilization)?	आपको पूरा विश्वास है ....4<br>Yes, completely confident I can.<br>हाँ, थोडा बहुत विश्वास है ....3<br>Yes, somewhat confident I can.<br>नहीं, मुझे बहुत विश्वास नहीं है<br>No, not very confident I can....2<br>नहीं, बिलकुल भी विश्वास नहीं हैं<br>No, not at all confident I can....1

Q. No.	QUESTIONS & FILTERS	CODING CATEGORIES
129	आपको कितना यकीन है की आप उन हरेक महिला को जिनके घर आप जाती हैं, जिसमें की दलित एवं मुस्लिम महिला भी शामिल हैं, को टीकाकरण कराने के लिए राज़ी कर सकती हैं. How confident are you that you can persuade every mother you visit, including a Dalit or Muslim mother, to get her child immunized?	आपको पूरा विश्वास है ....4<br>Yes, completely confident I can.<br>हाँ, थोडा बहुत विश्वास है ....3<br>Yes, somewhat confident I can.<br>नहीं, मुझे बहुत विश्वास नहीं है<br>No, not very confident I can...2<br>नहीं, बिलकुल भी विश्वास नहीं हैं<br>No, not at all confident I can...1
**Job Satisfaction & Attachment (कार्य संतुष्टि एवं लगाव)**<br>निर्देश: इस भाग में, मै आप से जानना चाहता हूँ की आप अपने नौकरी एवं इस काम को करने के लिए मिलने वाले समर्थन और मान्यताओं के बारे में क्या महसूस करती हैं. मैं पहले कथन को पढ़ूँगा. मेरे कथन पढ़ने के बाद आप बताएं की क्या आप उस कथन से "*पूरी तरह से सहमत, सहमत, असहमत और पूरी तरह से असहमत हैं*". याद रखें की आप के द्वारा दिए गए उतर गोपनीय है और इसे किसी से भी साझा (शेयर) नहीं किया जायेगा.<br>*Now I will ask how you feel about your job and the support and recognition you receive for doing your job. I will read a statement. After I read each statement, tell me if you strongly agree, agree, disagree or strongly disagree. Remember, your answers are confidential and will not be shared with anyone.*		
**Satisfaction (संतुष्टि)**		
130	मोटा-मोटी आप अपनी नौकरी से खुश हैं Overall, you are satisfied with your work.	पूरी तरह से सहमत Strongly agree....................................4<br>सहमत Agree...3<br>असहमत Disagree...2<br>पूरी तरह से असहमत Strongly disagree.............................1
**Overall Motivation (पूर्ण प्रोत्साहन)**		
131	आपको उस काम को करने में आनंद आता है जिसमे उत्तरदायित्व ज्यादा होता है. You enjoy work that requires great responsibilities	पूरी तरह से सहमत Strongly agree....................................4<br>सहमत Agree...3<br>असहमत Disagree...2<br>पूरी तरह से असहमत Strongly disagree.............................1
132	जब भी कोई मुश्किल काम आता है आप जल्दी हार मान जाती हैं. When faced with a difficult task you tend to give up quickly.	पूरी तरह से सहमत Strongly agree....................................4<br>सहमत Agree...3<br>असहमत Disagree...2<br>पूरी तरह से असहमत Strongly disagree.............................1
133	जब कभी आप अपने लक्ष्य को नहीं पाते हैं तो लक्ष्य को पाने के लिए आप एक बार फिर से जोर लगाती हैं. When you have not attained your goal, you exert yourself once again to attain the goal	पूरी तरह से सहमत Strongly agree....................................4<br>सहमत Agree...3<br>असहमत Disagree...2<br>पूरी तरह से असहमत Strongly disagree.............................1
134	आप प्राय: जितना काम करने का सोचती हैं उससे ज्यादा काम करती हैं. You usually do much more than you resolve to do.	पूरी तरह से सहमत Strongly agree....................................4<br>सहमत Agree...3<br>असहमत Disagree...2<br>पूरी तरह से असहमत Strongly disagree.............................1

Q. No.	QUESTIONS & FILTERS	CODING CATEGORIES
	**Sense of doing valuable work (अहम काम करने की भावना)**	
135	आप कुछ और करना चाहेंगी अगर आपको दूसरा काम मिल जाता है. You would do something else if you could get another job.	पूरी तरह से सहमत Strongly agree....................................4<br>सहमत Agree...3<br>असहमत Disagree...2<br>पूरी तरह से असहमत Strongly disagree............................1
136	आप अपने उप-स्वास्थ्य केंद्र के मिशन में विश्वास करती हैं. You believe in the mission of your health sub-centre.	पूरी तरह से सहमत Strongly agree....................................4<br>सहमत Agree...3<br>असहमत Disagree...2<br>पूरी तरह से असहमत Strongly disagree............................1
137	आप ये काम सिर्फ पैसे के लिए करती हैं. You only do this job for money.	पूरी तरह से सहमत Strongly agree....................................4<br>सहमत Agree...3<br>असहमत Disagree...2<br>पूरी तरह से असहमत Strongly disagree............................1
138	आप उन अवसरों से संतुष्ट हैं, जिसमें आपको आपकी क्षमताओं का उपयोग करने का मौका मिलता हैं. You are satisfied with the opportunity to use your abilities in this job.	पूरी तरह से सहमत Strongly agree....................................4<br>सहमत Agree...3<br>असहमत Disagree...2<br>पूरी तरह से असहमत Strongly disagree............................1
	**Perceived opportunity for growth & learning (विकास एवं सीखने हेतु कथित सुअवसर)**	
139	उप-स्वास्थ्य केंद्र की मीटिंग में जो जानकारियां मिलतीं हैं वो आपको बेहतर काम करने में मदद करती है. The information shared at the health sub-center meetings has helped you to do a better job.	पूरी तरह से सहमत Strongly agree....................................4<br>सहमत Agree...3<br>असहमत Disagree...2<br>पूरी तरह से असहमत Strongly disagree............................1
140	आप नई चीजें सीखने के लिए मिलने वाले अवसरों से संतुष्ट हैं. You are satisfied with the opportunities to learn new things.	पूरी तरह से सहमत Strongly agree....................................4<br>सहमत Agree...3<br>असहमत Disagree...2<br>पूरी तरह से असहमत Strongly disagree............................1
**Appreciation and Recognition (सराहना एवं मान्यता: अहम, मान्य, सराहनीय एवं समर्थित महसूस करना**		
141	समाज में प्रतिष्ठित स्थान पाना आपके लिए महत्त्वपूर्ण है. To attain a position of respect in the community is important to you.	पूरी तरह से सहमत Strongly agree....................................4<br>सहमत Agree...3<br>असहमत Disagree...2<br>पूरी तरह से असहमत Strongly disagree............................1
142	आप वैसे ग्रुप के सदस्य का आदर करते हैं जो की समुदाय में पहचाने एवं आदर किये जाते हैं. You admire the team member who gets respect and recognition in society.	पूरी तरह से सहमत Strongly agree....................................4<br>सहमत Agree...3<br>असहमत Disagree...2<br>पूरी तरह से असहमत Strongly disagree............................1
143	आपके ग्रुप के सदस्य आपका आदर करेंगे अगर आपका सम्बन्ध समुदाय के साथ बहुत अच्छा है. Your colleagues will respect you if you have good relations with the community members.	पूरी तरह से सहमत Strongly agree....................................4<br>सहमत Agree...3<br>असहमत Disagree...2<br>पूरी तरह से असहमत Strongly disagree............................1
144	आपके ग्रुप के कुछ सदस्य अपने कार्य के दायित्व को पूरा करने में उचित एवं पर्याप्त रूप से योगदान नहीं देते. Some members of your team do not	पूरी तरह से सहमत Strongly agree....................................4<br>सहमत Agree...3<br>असहमत Disagree...2

Q. No.	QUESTIONS & FILTERS	CODING CATEGORIES
	contribute appropriately & adequately in fulfilling work duties.	पूरी तरह से असहमत Strongly disagree.............................1
145	आपको अपने काम का उचित एवं नियमित वेतन मिलता है. You receive regular and reliable payment for your work.	पूरी तरह से सहमत Strongly agree....................................4<br>सहमत Agree..3<br>असहमत Disagree..2<br>पूरी तरह से असहमत Strongly disagree.............................1
146	ज्यादात्तर परिवार जंहा आप गृह भ्रमण करती हैं आपकी मदद की सराहना करते हैं. Most of the families you visit really appreciate your help.	पूरी तरह से सहमत Strongly agree....................................4<br>सहमत Agree..3<br>असहमत Disagree..2<br>पूरी तरह से असहमत Strongly disagree.............................1
147	आपको अपना काम बेहतर तरीके से करने के लिए जरुरी सुचनाये उपलब्ध नहीं हैं. You do not have access to all the information you need to do your job well.	पूरी तरह से सहमत Strongly agree....................................4<br>सहमत Agree..3<br>असहमत Disagree..2<br>पूरी तरह से असहमत Strongly disagree.............................1
148	आपके परिवार को आपके काम पर नाज है. Your family is proud of the work that you do.	पूरी तरह से सहमत Strongly agree....................................4<br>सहमत Agree..3<br>असहमत Disagree..2<br>पूरी तरह से असहमत Strongly disagree.............................1
**(एक समूह के रूप में काम करने के व्यवहार संबंधी परिणाम) Behavioural Outcomes: Works well as part of a team**<br>**निर्देश: अब मैं आपसे, आपके अपने साथी एवं सुपरवाइजर (पर्यवेक्षिका) के साथ संवाद के बारे में कुछ सवाल पूछूँगा. मैं एक कथन और उसके कुछ संभव उतर को पढ़ूँगा. कृपया आप बताएं की इनमें से कौन सा विकल्प आपके संवाद की सबसे अच्छी तरीके से व्याख्या करता है.**<br>***[Script:]*** *Now I will ask you a few questions about interacting with your co-workers and supervisor. I will read a statement and a set of responses. Please tell me which response you think most accurately describes your interaction.*		
149	आपके साथी आपसे उचित/जरुरी सुचना छुपातें हैं. Your colleagues hide appropriate information.	हमेशा Always...4<br>प्राय: Often...3<br>शायद ही कभी Rarely...2<br>कभी नहीं Never...1
150	आप जिन लोगों के साथ काम करते हैं वो एक दूसरे की मदद करते हैं. The people you work with help each other.	हमेशा Always...4<br>प्राय: Often...3<br>शायद ही कभी Rarely...2<br>कभी नहीं Never...1
151	जब आप के समूह के लोगों को कोई समस्या होती हैं तो आप उन्हें वो सारी बाते बतलाती हैं जो आप ने उस परिस्थिति में किया था.When another FLW encounters a problem, you share what you did when you encountered a similar problem.	हमेशा Always...4<br>प्राय: Often...3<br>शायद ही कभी Rarely...2<br>कभी नहीं Never...1
152	आप अकसर कितनी बार आशा से बातचीत करती हैं. How often do you interact with another ASHA?	प्रति दिनEvery day ……………………………………..1<br>सप्ताह में एक बार Once in a week……………………2<br>पंद्रह दिन में एक बार Once in a fortnight…………….3<br>महीने में एक बार Once in a month……………………4<br>शायद ही कभी Rarely……………………………………5<br>कभी नहीं Never………………………………………..6
153	आप अकसर कितनी बार आंगनवाडी सेविका से बातचीत करती हैं. How often do you interact with another AWW?	प्रति दिनEvery day ……………………………………..1<br>सप्ताह में एक बार Once in a week……………………2<br>पंद्रह दिन में एक बार Once in a fortnight…………….3

Q. No.	QUESTIONS & FILTERS	CODING CATEGORIES
		महीने में एक बार Once in a month……………………4<br>शायद ही कभी Rarely……………………………………5<br>कभी नहीं Never…………………………………….....6
154	आप अकसर कितनी बार ए न म से बातचीत करती हैं. How often do you interact with the ANM?	प्रति दिनEvery day ……………………………….1<br>सप्ताह में एक बार Once in a week……………………2<br>पंद्रह दिन में एक बार Once in a fortnight…………..3<br>महीने में एक बार Once in a month……………………4<br>शायद ही कभी Rarely……………………………………5<br>कभी नहीं Never…………………………………….....6
155	पिछली बार आपने कब किसी आशा/ आंगनवाड़ी सेविका से ऑफिस मीटिंग के अलावा बातचीत की थी. When was the last time you interacted with another AWW/ASHA other than at Official meetings?	पिछले महीने Last month…………………………….1<br>पिछले पंद्रह दिनों में Last fortnight……………………2<br>पिछले सप्ताह Last week……………………………….3<br>इसी सप्ताह This week………………………………….4<br>कभी नहीं Never………………………………………..5
156	पिछली बार आशा, आंगनवाड़ी सेविका के साथ आपने गृह भ्रमण कब किया था . When was the last time you did a joint home visit? (with another ASHA, AWW)	पिछले महीने Last month…………………………….1<br>पिछले पंद्रह दिनों में Last fortnight……………………2<br>पिछले सप्ताह Last week……………………………….3<br>इसी सप्ताह This week………………………………….4<br>कभी नहीं Never………………………………………..5
157	क्या आप प्रखंड स्तरीय मीटिंग में भाग लेती हैं? Do you attend Block Level meetings?	हर महीने Every month……………………………….1<br>कभी कभी Sometimes…………………………………..2<br>नहीं के बराबर Almost Never…………………………..3
158	क्या आप उप-स्वास्थ्य केंद्र के मीटिंग में भाग लेती हैं? Do you attend HSC meetings?	हर महीने Every month……………………………….1<br>कभी कभी Sometimes…………………………………..2<br>नहीं के बराबर Almost Never…………………………..3
**Behavioral Outcomes: Provides more Equitable Services (व्यवहार संबंधी परिणाम: समान सेवा प्रदान करना)**<br><br>**निर्देश: अब मै आपसे, आप काम कैसे करती हैं के बारे में पूछना चाहूँगा. *मैं एक कथन को पढ़ूँगा. कथन को पढ़ने के बाद, आप बताएं की आप इनमें से हरेक कार्य को हमेशा, प्रायः, शायद ही कभी, कभी भी नहीं करती है और ये आपके कार्यक्षेत्र के लिए लागू नहीं है.***<br>***[Script:]*** *Now I would like to ask a few questions about how you work. I will read a statement. After I read the statement, tell me whether you think you do each action Always, Often, Rarely or Never.*		
159	आप अपने क्षेत्र के सारे दलित गर्भवती महिलायों के घर भ्रमण करती हैं. You visit the homes of all the ***Dalit*** pregnant women in your catchment area.	हमेशा Always……………………………………………..1<br>प्राय: Often……………………………………………….2<br>शायद ही कभी Rarely……………………………………3<br>कभी नहीं Never…………………………………….....4<br>लागू नहीं (कोई दलित महिला नहीं है पोषक क्षेत्र में) Not applicable (no Dalit women in my catchment area)……………………………………………………5
160	आप अपने क्षेत्र के उन सारे दलित महिलायों के घर भ्रमण करती हैं जिनके छोटे बच्चें हैं. You visit the homes of all the ***Dalit*** women with young infants in your catchment area.	हमेशा Always……………………………………………..1<br>प्राय: Often……………………………………………….2<br>शायद ही कभी Rarely……………………………………3<br>कभी नहीं Never…………………………………….....4<br>लागू नहीं (कोई दलित महिला नहीं है पोषक क्षेत्र में) Not applicable (no Dalit women in my catchment area)……………………………………………………5

Q. No.	QUESTIONS & FILTERS	CODING CATEGORIES
161	आप अपने क्षेत्र के सारे मुसलिम गर्भवती महिलायों के घर भ्रमण करती हैं. You visit the homes of all the ***Muslim*** pregnant women in your catchment area	हमेशा Always..1<br>प्राय: Often……………………………………….2<br>शायद ही कभी Rarely…………………………….3<br>कभी नहीं Never………………………………....4<br>लागू नहीं (कोई दलित महिला नहीं है पोषक क्षेत्र में) Not applicable (no Dalit women in my catchment area)……………………………………………5
162	आप अपने क्षेत्र के उन सारे मुस्लिम महिलायों के घर भ्रमण करती हैं जिनके छोटे बच्चें हैं. You visit the home of all the ***Muslim*** women with young infants in your catchment area	हमेशा Always..1<br>प्राय: Often……………………………………….2<br>शायद ही कभी Rarely…………………………….3<br>कभी नहीं Never………………………………....4<br>लागू नहीं (कोई दलित महिला नहीं है पोषक क्षेत्र में) Not applicable (no Dalit women in my catchment area)……………………………………………5
**Behavioural Outcomes: Takes Initiative to solve problems (व्यवहार संबंधी परिणाम: समस्याओ को समाधान करने में पहल)**		
163	जब भी काम में कुछ गड़बड़ होता है आप तुरन्त समाधान ढूँढने लगती हैं. Whenever something goes wrong at work, you search for a solution immediately.	हमेशा Always..4<br>प्राय: Often..3<br>शायद ही कभी Rarely..................................2<br>कभी नहीं Never..1
164	आप तुरंत पहल करती हैं जबकि दुसरे नहीं करते हैं. You take initiative immediately, even when others do not	हमेशा Always..4<br>प्राय: Often..3<br>शायद ही कभी Rarely..................................2<br>कभी नहीं Never..1
165	आप उन चीजों को बिना बोलें करतीं है जिसे में आपके खास ध्यान की आवश्यकता होती है. You do things that need your attention without being asked or told to do so.	हमेशा Always..4<br>प्राय: Often..3<br>शायद ही कभी Rarely..................................2<br>कभी नहीं Never..1
166	जब भी आपको समस्या होती है आप मदद और सलाह ढूंढती हैं. When you encounter a problem you seek help or advice	हमेशा Always..4<br>प्राय: Often..3<br>शायद ही कभी Rarely..................................2<br>कभी नहीं Never..1
उप-स्वास्थ्य केंद्र के बैठक एवं प्रोत्साहन का कथित मूल्य: **Perceived value of the HSC meetings**<br>**निर्देश: अब में आपसे पूछना चाहूँगा की आप उप-स्वास्थ्य केंद्र की बैठक के बारे में क्या महसूस करती हैं? *मैं एक कथन को पढूँगा. कथन को पढ़ने के बाद, आप बताएं की क्या आप नीचे दिए गए एहसास को हमेशा, प्रायः, शायद ही कभी, या कभी भी नहीं महसूस करती है.***<br>***[Script:]*** *Now I would like to ask a few questions about how you feel about the HSC meeting. I will read a statement. After I read the statement, tell me whether you feel that way Always, Often, Rarely or Never.*		
167	आप उप-स्वास्थ्य केंद्र के मीटिंग को सुखद पाती हैं. You find the HSC meeting very pleasant	हमेशा Always..4<br>प्राय: Often..3<br>शायद ही कभी Rarely..................................2<br>कभी नहीं Never..1
168	आप उप-स्वास्थ्य केंद्र की मीटिंग को तनाव पूर्ण पाती है. You find the HSC meeting very stressful.	हमेशा Always..4<br>प्राय: Often..3<br>शायद ही कभी Rarely..................................2<br>कभी नहीं Never..1

Q. No.	QUESTIONS & FILTERS	CODING CATEGORIES
169	आप उप-स्वास्थ्य केंद्र की मीटिंग सूचनाप्रद पाती है. You find the HSC meeting very informative.	हमेशा Always....4<br>प्राय: Often....3<br>शायद ही कभी Rarely....2<br>कभी नहीं Never....1
170	आप उप-स्वास्थ्य केंद्र की मीटिंग बहुत ही अव्यवस्थित पाती है. You find the HSC meeting much unorganized.	हमेशा Always....4<br>प्राय: Often....3<br>शायद ही कभी Rarely....2<br>कभी नहीं Never....1
171	उप-स्वास्थ्य केंद्र की मासिक बैठक आपको कठिन काम करने के लिए प्रेरित करती है. Monthly HSC meetings inspire you to work harder.	हमेशा Always....4<br>प्राय: Often....3<br>शायद ही कभी Rarely....2<br>कभी नहीं Never....1
**ANM Leadership (ए न ऍम नेतृत्व)**<br>*निर्देश अगले प्रश्नों के समूह में मैं आपसे उन विषय के बारे में पूछूँगा जो कार्य आपके उप-स्वास्थ्य केंद्र की ए न म करती हैं. कृपया आप बताएं की क्या वो करती हैं हाँ, या नहीं, वो नहीं करती हैं.*<br>***[Script:]*** *The next set of questions asks about things the ANM of your health sub-centre does, tell me whether yes your ANM does each thing, or no they do not*		
172	आपकी अ. न. म. रिकॉर्ड और रिपोर्ट्स के सत्यता की समीक्षा/जाँच करती. Your ANM Reviews your records and reports for accuracy?	हाँ Yes.... 1<br>नहीं No....2
173	आपकी अ. न. म. गृह भ्रमण के दौरान आपका अवलोकन करती है. Your ANM Observes you during home visits	हाँ Yes.... 1<br>नहीं No....2
174	आपकी अ. न. म. आपके प्रदर्शन पर प्रतिक्रिया (फीडबैक) देती हैं. Your ANM Provides you with direct feedback about your performance	हाँ Yes.... 1<br>नहीं No....2
175	आप जब किसी समस्या का सामना करती हैं तो आपकी अ. न. म. आपकी मदद करती हैं. Your ANM Assists you with problems or difficulties you are facing	हाँ Yes.... 1<br>नहीं No....2
**आप ऐसा महसूस करती हैं की आपके उप-स्वास्थ्य केंद्र की ए न म:**<br>*अब मैं आपसे जानना चाहूँगा की आप ये महसूस करती हैं की किसी विशेष काम को करने की जिम्मेवारी आपके अ. न. म. की है. मेरे प्रत्येक कथन के पढ़ने के बाद आप बतायें की क्या आप उस कथन से "पूरी तरह से सहमत, सहमत, असहमत और पूरी तरह से असहमत हैं".*<br>***[Script]:*** *I would like to know if you feel that it is your ANM's responsibility to do a particular thing. After I read each statement, tell me whether you Strongly Agree, Agree, Disagree or Strongly Disagree it is your ANM's responsibility.*		
176	ग्रुप के सदस्यों के प्रदर्शन का दायित्तव लेती है. It is your ANM's responsibility to Take responsibility for the performance of the group members	पूरी तरह से सहमत Strongly agree....4<br>सहमत Agree....3<br>असहमत Disagree....2

Q. No.	QUESTIONS & FILTERS	CODING CATEGORIES
		पूरी तरह से असहमत Strongly disagree……………………….1
177	ग्रुप सदस्य को प्रेरित या प्रोत्साहित करती है. It is your ANM's responsibility to Inspire or motivate the group members	पूरी तरह से सहमत Strongly agree……………………………4<br>सहमत Agree……………………………………………………3<br>असहमत Disagree…………………………………………….2<br>पूरी तरह से असहमत Strongly disagree……………………….1
178	ग्रुप के सदस्यों को समर्थ (सशक्त) बनाती हैं. It is your ANM's responsibility to Empower the group members	पूरी तरह से सहमत Strongly agree……………………………4<br>सहमत Agree……………………………………………………3<br>असहमत Disagree…………………………………………….2<br>पूरी तरह से असहमत Strongly disagree……………………….1
179	ग्रुप के सदस्यों को नेतृत्तव प्रदान करती है. It is your ANM's responsibility to provide leadership to group members	पूरी तरह से सहमत Strongly agree……………………………4<br>सहमत Agree……………………………………………………3<br>असहमत Disagree…………………………………………….2<br>पूरी तरह से असहमत Strongly disagree……………………….1
180	ग्रुप के सदस्यों के बीच सभी को साथ लेकर चलने के व्यवहार (समावेशता/समग्रता) को प्रोत्साहित करती है. It is your ANM's responsibility to Foster inclusiveness among the group members	पूरी तरह से सहमत Strongly agree……………………………4<br>सहमत Agree……………………………………………………3<br>असहमत Disagree…………………………………………….2<br>पूरी तरह से असहमत Strongly disagree……………………….1
181	ग्रुप के सदस्यों के बीच मनमुटाव का समाधान करती है. It is your ANM's responsibility to resolve conflicts among the group members	पूरी तरह से सहमत Strongly agree……………………………4<br>सहमत Agree……………………………………………………3<br>असहमत Disagree…………………………………………….2<br>पूरी तरह से असहमत Strongly disagree……………………….1

Scenarios for Behavioral Outcomes

निर्देश: अब मैं आपके सामने अलग-अलग सिनेरियो और कई अलग-अलग विकल्पों ***को रखूँगा. प्रत्येक कथन के पढ़ने के बाद आप अपने शब्दों में बतायें की आप क्या सोचती है की उन अलग-अलग परिस्थितियों में आप क्या करेगी?***

[Script:] Now I will present you with several different scenarios and several different options for what you would do in each situation. After I read each statement, tell me in your own words what you think you would do in each situation.

Q. No.	QUESTIONS & FILTERS	CODING CATEGORIES
182	कल्पना करे की अगर आपका बच्चा बीमार हैं जिसके कारण आप कुछ दिनों तक काम करने में सक्षम नहीं है तो आप ऐसे अवस्था में क्या करेंगी?<br>Imagine your own child gets sick and you cannot work for a few days, what do you do?	**अतिरिक्त निर्देश (जोर से ना पढ़े):** कृप्या विकल्पों को ना पढ़े? आशा आंगनवाडी सेविका को खुली प्रतिक्रिया देने दें, उसके बाद उस विकल्प पर गोला करें जो आशा/आंगनवाड़ी सेविका के दिए गए जवाब से सबसे ज्यादा मिलता जुलता है. इसका सिर्फ एक ही जवाब हो सकता है. **[Additional Instructions (do not read aloud):** DO NOT READ OPTIONS. Allow for free response from the ASHA/AWW, then circle the response option that most closely matches what the ASHA/AWW says. There can only be ONE response.]<br><br>घर पर रहेगी और काम पर नहीं जायेगी. Stay at home and don't show up for work…………………………………..1

		अपने समूह के आशा एवं आंगनवाड़ी सेविका से मदद के लिए कहती हैं. Ask another ANW or ASHA of your group for help with your work……………………………………….2<br><br>अ.न.म से सलाह लेगी की क्या करना हैं Ask the ANM for advice on what to do……………………………………….3
183	मान लीजिये की इस महीने में आपको बहुत ही ज्यादा गृह भ्रमण करने की आवश्यकता है. आप कौन से घर का भ्रमण पहले करेंगी.<br>Imagine you have a very busy month of home visits that need to be conducted. Who would you visit first?	**अतिरिक्त निर्देश (जोर से ना पढ़े):** कृप्या विकल्पों को ना पढ़े? आशा आंगनवाडी सेविका को खुली प्रतिक्रिया देने दें, उसके बाद उस विकल्प पर गोला करें जो आशा/आंगनवाडी सेविका के दिए गए जवाब से सबसे ज्यादा मिलता जुलता है. इसका सिर्फ एक ही जवाब हो सकता है. **[Additional Instructions (do not read aloud):** DO NOT READ OPTIONS. Allow for free response from the ASHA/AWW, then circle the response option that most closely matches what the ASHA/AWW says. There can only be ONE response.]<br><br>आप उस औरत के घर का भ्रमण पहले करेंगी जिसके घर में एक सप्ताह से कम का नवजात बच्चा है. You would visit the women with newborns within first week of birth…………………………………………………………1<br><br>आप उस गर्भवती महिला के घर जाएँगी जिसका प्रसव तुरंत होने वाला है. You would visit the pregnant women about to deliver soon…………………………………………………2<br><br>जिस घर में आसानी से पंहुचा जा सकता है उस घर का भ्रमण पहले करेंगी. You would visit the houses of those You can reach most easily………………………………………….3<br><br>आप उस महिला के घर पहले जाएँगी जो आपकी बातों को ज्यादा मानेगी. You would visit the women who are most likely to listen to You……………………………………………….4<br><br>आप महिला के घर पहले जाएँगी जिसके साथ आपके सम्बन्ध अच्छे हैं. You would visit the women who You have a good relationship with…………………………………………..5<br><br>आप उस घर का भ्रमण पहले करेंगी जिसके घर का आप या कोई आशा और आंगनवाडी सेविका पहले भ्रमण नहीं किया है. You would visit those women who You know haven't been visited by another ASHA or AWW in the last month…………………………………………………………6<br><br>आप उस महिला के घर पहले जायेंगी जो की आपकी सेवाओं का लाभ नहीं ले रही हैं. You would visit those women who You know haven't been using services………………………….7<br><br>अन्य other ______________________________<br>(स्पष्ट करें/ specify)

184	कल्पना कीजिये की आप वैसी महिला के घर जिनका चार-पाँच महीना का बच्चा है, को छह महीने के बाद बच्चों को दी जाने वाली पूरक आहार के बारे में परामर्श देने गयी हैं. जब आप उस महिला को परामर्श देने लगती हैं तो उसकी सास आप की बातों से असहमति जताते हुए ये कहती है की जब तक बच्चा नौ महीने का ना हो जाये उसे कुछ नहीं देना है. ऐसे में आप क्या करती हैं जब सास आपकी के बातों से सहमत नहीं होती है? Imagine you visit the house of a mother with a 4 or 5 month old to counsel on complementary feeding starting at 6 months of age. *But* when you start counseling the mother, her mother-in-law disagrees with you by saying the mother should wait until the baby is 9 months old. What do you do when the mother-in-law disagrees with you?	**अतिरिक्त निर्देश (जोर से ना पढ़े):** कृप्या विकल्पों को ना पढ़े? आशा आंगनवाडी सेविका को खुली प्रतिक्रिया देने दें, उसके बाद उस विकल्प पर गोला करें जो आशा/आंगनवाड़ी सेविका के दिए गए जवाब से सबसे ज्यादा मिलता जुलता है. इसका सिर्फ एक ही जवाब हो सकता है. **[Additional Instructions (do not read aloud):** DO NOT READ OPTIONS. Allow for free response from the ASHA/AWW, then circle the response option that most closely matches what the ASHA/AWW says. There can only be ONE response.]<br><br>आप परामर्श देती हैं और चली जाती है, आप ने जानकारी दे दी, अब आप और कुछ नहीं कर सकती है. Finish your counseling and leave, you gave the information, there is nothing else you can do ……………………………………………………1<br><br>आप वापस उस घर में जाएँगी जब उस महिला की सास घर पर नहीं होगी. Go back to the house when you know the mother-in-law is not there....…………………………………2<br><br>अगले गृह भ्रमण के दौरान उस महिला के सास से बात करने के लिए, आप दूसरी आशा और आंगनवाडी सेविका या अ.न.म को लेकर आयगी. Bring another FLW or the ANM with you to the next visit to talk to the Mother-in-law…………………3<br><br>आप जानकारी एवं अपना मोबाइल नंबर छोड़ कर जाएँगी ताकि कोई सवाल होने पर वो महिला आपसे संपर्क कर सके. Leave behind information and your number for the mother in case she has questions……………………………………………..4<br><br>आप उस महिला के पति से बात करेंगी और उन्हें बोलेंगी की वो अपने माँ से बात करें. You talk to her husband to convince his mother……………………………………………………..5<br><br>अन्य other_______________________________________<br>(स्पष्ट करें/ specify)
185	कल्पना कीजिय की आप किसी नवजात बच्चा के घर में उसकी माँ को टीकाकरण करने की महत्ता पर परामर्श के लिए भ्रमण कर रही हैं, लेकिन वो महिला आपकी सलाह मानने एवं सेवा लेने से इनकार कर देती है. ऐसे में आप क्या करती है? Imagine you visit the home of a new born in order to counsel her mother on the importance of immunization, but the woman refuses to listen to your counseling or receive your services. What do you do?	**अतिरिक्त निर्देश (जोर से ना पढ़े):** कृप्या विकल्पों को ना पढ़े? आशा आंगनवाडी सेविका को खुली प्रतिक्रिया देने दें, उसके बाद उस विकल्प पर गोला करें जो आशा/आंगनवाड़ी सेविका के दिए गए जवाब से सबसे ज्यादा मिलता जुलता है. इसका सिर्फ एक ही जवाब हो सकता है. **[Additional Instructions (do not read aloud):** DO NOT READ OPTIONS. Allow for free response from the ASHA/AWW, then circle the response option that most closely matches what the ASHA/AWW says. There can only be ONE response.]<br><br>आप चली जाती हैं क्योंकि आप कुछ कर नहीं सकती. Leave, there is nothing you can do……………………………….1<br><br>कुछ समय के बाद उस घर पर फिर जाती हैं एक और कोशिश करने के लिए. Go back to the house after some time to try again……………………………………………………..2

		उस महिला को समझाने में मदद करने के लिए अगले भ्रमण के दौरान दुसरे आशा/आंगनवाडी सेविका या अ.न.म. को लेकर आती है. Bring another ASHA/AWW or the ANM with you to the next visit for support in convincing the woman to listen……………………………………………………..3<br><br>आप जानकारी एवं अपना मोबाइल नंबर छोड़ कर जाति हैं ताकि महिला अगर अपना मन बदले तो या कोई सवाल हो तो वो महिला आप से संपर्क कर सके. Leave behind information and your number in case the woman changes her mind or has questions………………………………………………4<br><br>आप पहले उसके पति या सास से बात करेंगी. Talk to the husband or mother-in-law first……………………………5<br><br>अन्य other_______________________________________<br>(स्पष्ट करें/ specify)
186	कल्पना कीजिये की आपके प्रखंड में आई. एफ. ए टेबलेट्स का स्टॉक खत्म हो गया है. ऐसे में आप क्या करती है? Imagine there is a stock-out of IFA tablets in your block. What do you do?	**अतिरिक्त निर्देश (जोर से ना पढ़े):** कृप्या विकल्पों को ना पढ़े? आशा आंगनवाडी सेविका को खुली प्रतिक्रिया देने दें, उसके बाद उस विकल्प पर गोला करें जो आशा/आंगनवाड़ी सेविका के दिए गए जवाब से सबसे ज्यादा मिलता जुलता है. इसका सिर्फ एक ही जवाब हो सकता है. **[Additional Instructions (do not read aloud):** DO NOT READ OPTIONS. Allow for free response from the ASHA/AWW, then circle the response option that most closely matches what the ASHA/AWW says. There can only be ONE response.]<br><br>कुछ नहीं कर सकती, टेबलेट्स के सप्लाई का नियंत्रण आपके हाथ में नहीं है. Nothing, supply of tablets are out of your control………………………………………………………1<br><br>आप अ.न.म से उनकी सुझाव/मदद के लिए उनसे बात करती है. Talk to your ANM to see if she can help or have suggestions…………………………………………………2<br><br>आप प्रखंडस्तरीय पदाधिकारी को रिपोर्ट करती हैं. Report it to the Block level officials……………………………………3<br><br>आप लाभार्थी को टेबलेट्स खरीदने के लिए बोलती हैं. Ask the beneficiary to buy IFA……………………………………4<br><br>अन्य other_______________________________________<br>(स्पष्ट करें/ specify)
187	मान लीजिये की आप अपने रिश्तेदार के घर गई हैं जो की आपके कार्यक्षेत्र से काफी दूर है और उसी समय आपको एक ऐसी महिला का फ़ोन आता है जिसका प्रसव होने वाला है और नाजुक अवस्था में है. ऐसे अवस्था में आप क्या करती हैं? Suppose you have gone to visit you relative which is quite far from your work area and during that time you receive a call from a pregnant women	**अतिरिक्त निर्देश (जोर से ना पढ़े):** कृप्या विकल्पों को ना पढ़े? आशा आंगनवाडी सेविका को खुली प्रतिक्रिया देने दें, उसके बाद उस विकल्प पर गोला करें जो आशा/आंगनवाड़ी सेविका के दिए गए जवाब से सबसे ज्यादा मिलता जुलता है. इसका सिर्फ एक ही जवाब हो सकता है. **[Additional Instructions (do not read aloud):** DO NOT READ OPTIONS. Allow for free response from the ASHA/AWW, then circle the response option that most

	who is about to deliver a baby and is in critical situation. What would you do?	closely matches what the ASHA/AWW says. There can only be ONE response.]<br>आप कुछ नहीं कर सकती क्योंकि आप वापस लौट नहीं सकती. You can't do anything because you can't come back………………………………………………………….1<br><br>आप अपने ग्रुप के सदस्यों को सूचित करेंगी और उस महिला को मदद पहुंचाने के लिए बोलेंगी. You will inform your group member and request them to help her out…………………2<br><br>आप उस महिला को नजदीक के सरकारी अस्पताल में जाने को बोलेंगी. You will tell that woman to visit nearest Government hospital………………………………………….3<br><br>आप उस महिला को नजदीक के किसी निजी अस्पताल में जाने को बोलेंगी. You will tell that woman to visit nearest private hospital……………………………………………………..4<br><br>आप किसी अप्रशिक्षित सेवा प्रदाता को उनसे मिलने के लिए बोलेगी You will arranged untrained provider to visit her……………………………………………………………..5<br><br>आप स्वास्थ्य सेवा प्रदाता का नाम एवं नंबर उस महिला को देकर उससे मिलने को बोलेंगी You will provide name and contact number of health provider and ask her to visit…………………………………………………………..6<br><br>अन्य other____<br>(स्पष्ट करें/ specify)

इस भाग के सवाल केवल उन आशा एवं आंगनवाडी सेविकायों के लिए है जो की टी. बी. जी. आई. के इंटरवेंशन के अंतर्गत आते हैं. The following section contains questions for those ASHAs and AWWs who received the TBGI INTERVENTION ONLY.

टी. बी. जी. आई. का कथित मूल्य (Perceived Value of the TBGI Intervention)

निर्देश: अब मैं आपसे केयर इंडिया इंटरवेंशन के उस पक्ष के बारे में पूछना चाहूँगा जिससे आपका काम को आसान हो गया है और आपको काम करने के लिए प्रेरित करता है. ***[Script:]*** *I would now like to ask you a few questions about what aspects of the CARE intervention has made your work easier and has most motivated you.*

Q. No.	QUESTIONS & FILTERS	CODING CATEGORIES
188	इनमें में से कौन सी प्रक्रिया ने आपके काम को आसान बना दिया है. Which is the most important process that has made your work easier?	विभिन्न सूचक के लिए निर्धारित टारगेट............................ ☐<br>Fixed target for various indicators<br><br>एक साथ काम करना.. ☐<br>Working together.<br><br>नियमित मासिक बैठक.. ☐<br>Regular monthly meeting.<br><br>उपलब्धियों की नियमित समीक्षा.. ☐<br>Regular review of achievement.
189	इनमें में से कौन सी प्रक्रिया ने आपके काम को आसान बना दिया है. Which is the **SECOND** most important process that has made your work easier?	विभिन्न सूचक के लिए निर्धारित टारगेट............................ ☐<br>Fixed target for various indicators<br><br>एक साथ काम करना.. ☐<br>Working together.<br><br>नियमित मासिक बैठक.. ☐<br>Regular monthly meeting.<br><br>उपलब्धियों की नियमित समीक्षा.. ☐<br>Regular review of achievement.
190	इसमें से कौन से फैक्टर (घटक) ने आपके ज्यादा काम करने की इच्छा को प्रभावित किया है. Which is the most important factor that has affected your motivation to work?	विभिन्न सूचक के लिए नियत टारगेट.................................. ☐<br>Fixed target for various indicators.<br><br>एक साथ टीम के रूप में काम करना...................................... ☐<br>Team work.<br><br>गैर-मौद्रिक प्रोत्साहन.. ☐<br>Non-cash incentives.<br><br>सरकार के वरीय पदाधिकारी द्वारा दिए गए सर्टिफिकेट......... ☐<br>Certificates given by the senior government officials.<br><br>उप-स्वास्थ्य केंद्र के मासिक बैठक में काम की नियमित समीक्षा<br>Regular review of work at HSC meeting.................................. ☐
191	इसमें से कौन से फैक्टर (घटक) ने आपके ज्यादा काम करने की इच्छा को प्रभावित किया है. Which is the **SECOND** most important factor that has affected your motivation to work?	विभिन्न सूचक के लिए नियत टारगेट.................................. ☐<br>Fixed target for various indicators.<br><br>एक साथ टीम के रूप में काम करना...................................... ☐<br>Team work.

		गैर-मौद्रिक प्रोत्साहन.. ☐<br>Non-cash incentives.<br><br>सरकार के वरीय पदाधिकारी द्वारा दिए गए सर्टिफिकेट......... ☐<br>Certificates given by the senior government officials.<br><br>उप-स्वास्थ्य केंद्र के मासिक बैठक में काम की नियमित समीक्षा<br>Regular review of work at HSC meeting.................................. ☐
**Perceived Value of Non-Cash Incentives**<br>अब मैं आपसे नॉन-कैश (गैर-नकदी) प्रोत्साहन से सम्बंधित कुछ प्रश्न करना चाहूँगा.<br>***[Script:]*** *Now I would like to ask few questions regarding the non- cash incentives.*		
192	क्या आप जानते हैं की आपको प्रोत्साहन क्यों मिल रहा है? Do you know why are you getting the non-cash incentive? Please tell me in your own words why you think you are getting the non-cash incentive.	**[Additional Instructions (do not read aloud):** DO NOT READ OPTIONS. Allow for free response from the ASHA/AWW, then circle all of the responses options that most closely match what the ASHA/AWW says. There may be multiple responses.]<br><br>अच्छा काम करने के लिए To work well.......................................A<br><br>सारे लाभार्थियों तक पहुँचने के लिए To get reach of all the beneficiaries..B<br><br>बैठक को उचित तरीके से करने के लिए To conduct the meeting in proper manner..C<br><br>ग्रुप के सदस्यों अच्छा कम कर सके उसमे मदद करने के लिए To help the team member to work well.......................................D<br><br>समय पर टारगेट को पूरा करने के लिए. To achieve the target on time...E<br><br>अन्य Other ____X<br>(स्पष्ट करें/ specify)
193	क्या आप नॉन-कैश (गैर-नकदी) प्रोत्साहन से संतुष्ट हैं? Are you satisfied with the non-cash incentives:	हाँ Yes... 1<br>नहीं No... 2
194	आप नॉन-कैश (गैर-नकदी) प्रोत्साहन का उपयोग कैसे करती हैं? **Please tell me in your own words,** how do you utilize the Non-cash incentives:	**अतिरिक्त निर्देश (जोर से ना पढ़े):** कृप्या विकल्पों को ना पढ़े? आशा आंगनवाडी सेविका को खुली प्रतिक्रिया देने दें, उसके बाद उस विकल्प पर गोला करें जो आशा/आंगनवाड़ी सेविका के दिए गए जवाब से सबसे ज्यादा मिलता जुलता है. इसका सिर्फ एक ही जवाब हो सकता है. **[Additional Instructions (do not read aloud):**DO NOT READ OPTIONS. Allow for free response from the ASHA/AWW, then circle all of the responses options that most closely match what the ASHA/AWW says. There may be multiple responses.]<br><br>घर के कामों के लिए For household purpose..............................A<br><br>बेटी के शादी के लिए For daughters marriage.............................B<br><br>किसी और को उपहार देने के लिए As a gift to someone............C<br><br>किस काम का नहीं No use...D<br><br>अन्य Other ____X

		(स्पष्ट करें/ specify)
195	अगर मान लीजिये कि किसी कारणवश प्रोत्साहन अचानक से बंद हो जाये, तो क्या आप फिर भी टारगेट को समय पर पूरा करने की कोशिश करेंगी? Let us assume that due to some reason, non-cash incentives would stop all of sudden; will you still try to achieve the target on time?	हाँ Yes.. 1<br>नहीं No.. 2
196	अगर हाँ, तो बिना किसी प्रोत्साहन के टारगेट को पूरा करने के लिए आप क्यों अतिरिक्त मेहनत करेंगी. If yes, without any incentive why would you put in extra effort to still achieve the target on time? Please tell me in your own words why you would still try to achieve the target on time.	**अतिरिक्त निर्देश (जोर से ना पढ़े):** कृप्या विकल्पों को ना पढ़े? आशा आंगनवाडी सेविका को खुली प्रतिक्रिया देने दें, उसके बाद उस विकल्प पर गोला करें जो आशा/आंगनवाड़ी सेविका के दिए गए जवाब से सबसे ज्यादा मिलता जुलता है. इसका सिर्फ एक ही जवाब हो सकता है. **[Additional Instructions (do not read aloud):** DO NOT READ OPTIONS. Allow for free response from the ASHA/AWW, then circle all of the responses options that most closely match what the ASHA/AWW says. There may be multiple responses.]<br><br>हम लोग बेहतर करण के लिए काम रहे है.................................A<br>We are working for better cause.<br><br>यह मेरा कर्तव्य/ दायित्व हैं ....B<br>This is my duty/ responsibilities.<br>प्रोत्साहन मेरे लिए कोई मायने नहीं रखता..................................C<br>Incentive does not matter for me.<br>समूह कार्य के लिए..D<br>Team work.<br><br>अन्य Other ____X<br>(स्पष्ट करें/ specify)
197	नॉन-कैश (गैर-नकदी) प्रोत्साहन आपके परिवार वाले को इस बात के लिए सहमत करने में मदद कर रहा हैं की आप और ज्यादा काम करे Non-cash incentive is helping you to convince your family to allow you to work more?	पूरी तरह से सहमत Strongly agree.....................................4<br>सहमत Agree...3<br>असहमत Disagree..2<br>पूरी तरह से असहमत Strongly disagree.............................1
198	आपके परिवार में प्रोत्साहन मुख्य रूप से किसको प्रभावित करता है? Who does the non-cash incentive impact the most in your family?	बच्चे Children..1<br>पति Husband...2<br>परिवार के अन्य सदस्य Other family members.................3
199	प्रोत्साहन ने किस तरह से आपके परिवारवालो को प्रभावित किया है? Please tell me in your own words, what kind of impacts has the non-cash incentive had on your family members?	**अतिरिक्त निर्देश (जोर से ना पढ़े):** कृप्या विकल्पों को ना पढ़े? आशा आंगनवाडी सेविका को खुली प्रतिक्रिया देने दें, उसके बाद उस विकल्प पर गोला करें जो आशा/आंगनवाड़ी सेविका के दिए गए जवाब से सबसे ज्यादा मिलता जुलता है. इसका सिर्फ एक ही जवाब हो सकता है. **[Additional Instructions (do not read aloud):**DO NOT READ OPTIONS. Allow for free response from the ASHA/AWW, then circle all of the responses options that most closely match what the ASHA/AWW says. There may be multiple responses.]

		ज्यादा आदर मिलता है क्योंकि नॉन-कैश प्रोत्साहन पैसे से ज्यादा असरदार है. Getting more respect because it works more than money……………………………………………………………………………………A<br>यह आपके परिवार के सदस्यों के चेहरे पर ख़ुशी लाती है. It brings smile to the face of family member……………………………B<br>परिवारवालो से घर का काम काज ख़त्म करने में ज्यादा मदद मिलती है Getting more help from family member to finish household chore ……………………………………………………………………C<br>मेरे काम का आदर/मूल्य बढ़ गया है. Respect/value has increased for my work……………………………………………………………D<br>अन्य Other ____X<br>(स्पष्ट करें/ Specify)
200	नॉन-कैश (गैर-नकदी) प्रोत्साहन ने आपके परिवार का आपके और आपके काम के प्रति दृष्टिकोण को बदल दिया है. Non-cash incentives have changed the perception of your family towards you and your work.	पूरी तरह से सहमत Strongly agree……………………………….4<br>सहमत Agree………………………………………………………………….3<br>असहमत Disagree………………………………………………………..2<br>पूरी तरह से असहमत Strongly disagree………………………..1
201	अगर आप सहमत है तो कृप्या व्याख्या करें, कैसे? If agree, could you explain, How?	**अतिरिक्त निर्देश (जोर से ना पढ़े):** कृप्या विकल्पों को ना पढ़े? आशा आंगनवाडी सेविका को खुली प्रतिक्रिया देने दें, उसके बाद उस विकल्प पर गोला करें जो आशा/आंगनवाड़ी सेविका के दिए गए जवाब से सबसे ज्यादा मिलता जुलता है. इसका सिर्फ एक ही जवाब हो सकता है. **[Additional Instructions (do not read aloud):** Allow for free response from the ASHA/AWW, then try to capture the main points of what the ASHA/AWW says. If anything is unclear you can ask the ASHA/AWW to explain further.]<br>______________________________<br>______________________________<br>______________________________<br>______________________________<br>______________________________<br>______________________________<br>______________________________
